# Network analysis to identify symptoms clusters and temporal interconnections in oncology patients

**DOI:** 10.1038/s41598-022-21140-4

**Published:** 2022-10-12

**Authors:** Elaheh Kalantari, Samaneh Kouchaki, Christine Miaskowski, Kord Kober, Payam Barnaghi

**Affiliations:** 1 Centre for Vision, Speech and Signal Processing (CVSSP), University of Surrey, Guildford, UK; 2 Department of Physiological Nursing, University of California San Francisco, San Francisco, CA USA; 3 Department of Brain Sciences, Imperial College London, London, UK; 4 UK Dementia Research Institute Care Research and Technology Centre, Imperial College London, London, UK

**Keywords:** Breast cancer, Chemotherapy, Pain management, Computer science

## Abstract

Oncology patients experience numerous co-occurring symptoms during their treatment. The identification of sentinel/core symptoms is a vital prerequisite for therapeutic interventions. In this study, using Network Analysis, we investigated the inter-relationships among 38 common symptoms over time (i.e., a total of six time points over two cycles of chemotherapy) in 987 oncology patients with four different types of cancer (i.e., breast, gastrointestinal, gynaecological, and lung). In addition, we evaluated the associations between and among symptoms and symptoms clusters and examined the strength of these interactions over time. Eight unique symptom clusters were identified within the networks. Findings from this research suggest that changes occur in the relationships and interconnections between and among co-occurring symptoms and symptoms clusters that depend on the time point in the chemotherapy cycle and the type of cancer. The evaluation of the centrality measures provides new insights into the relative importance of individual symptoms within various networks that can be considered as potential targets for symptom management interventions.

## Introduction

Multiple co-occurring symptoms are the rule, rather than the exception, in oncology patients^1–3^. Over the past four to five decades, symptom scientists focused on an evaluation of the occurrence or severity of single symptoms (e.g., fatigue, pain, nausea) or reported the overall symptom experience of cancer patients. In 2001, the idea of a “symptom cluster” appeared in the oncology literature^4,5^. While the definition of a symptom cluster is evolving^3^, the major characteristics of a symptom cluster include: two or more concurrent symptoms, a relatively stable group of symptoms, independence from other clusters, and a common underlying mechanism(s). As noted in a report from an expert panel^3^, a growing body of research has identified symptom clusters in oncology patients and in patients with other chronic conditions (e.g., congestive heart failure). Most of these studies have identified symptom clusters using either cluster analysis or exploratory factor analysis (EFA)^6^. Using these analytic approaches, the three most common symptom clusters with similar characteristics were labelled: gastrointestinal, psychological, and a cluster that included pain, fatigue, sleep disturbance, and depression. While progress is being made in understanding the relationships among symptoms within a cluster, one of the major limitations of the analytical approaches used to date is that each symptom cluster is created as a silo. Techniques like cluster analysis and factor analysis do not allow for an evaluation of the interactions among symptom clusters. For example, the relationship between a psychological and gastrointestinal cluster or the relationships between and among symptoms in one cluster versus another.

Equally important in the field of symptom cluster research is an evaluation of the stability of symptom clusters over time (e.g., during a course of chemotherapy). While fewer in number, findings from the majority of the longitudinal studies of symptom clusters from our research team^7–9^ and others^10–12^ suggest that symptom clusters in oncology patients remain relatively stable over time. The “stability” analysis of the clusters was done primarily through a count of the number of symptoms that remained the same within an individual cluster (e.g., psychological symptom cluster) over time. Due to the inherent limitations in the analytic procedures that were used in the previous studies, no evaluation was done of how the strength of the relationships between and among the symptoms within a cluster changed over time and how the relationships between and among the symptom clusters changed over time.

Analytic techniques such as network analysis (NA) allow for a more comprehensive evaluation of the relationships between and among symptoms within a cluster and across various symptom clusters. For example, in our recent work^13^, we used NA to examine the relationships among multiple co-occurring symptoms and symptom clusters. This cross-sectional study, provided the first evidence to suggest that connections between and among symptoms differ depending on the symptom dimension (i.e., occurrence, severity, distress) used to create the network. We noted that longitudinal studies are needed that use methods like NA to evaluate for changes in symptom clusters over time.

In this paper, we present an analytical model to represent the relationships among individual symptoms and symptom clusters over a total of six time points across two cycles of chemotherapy (CTX). In addition, we analyse the clusters across time for one type of cancer (i.e., breast) to provide new insights into changes in the relationships between and among symptoms and symptom clusters in patients with a specific cancer diagnosis. The findings from this work can be used by clinicians to perform comprehensive assessments of the symptom experience of oncology patients and offer more tailored interventions. Future research in this area should focus on the translation of this type of evidence into clinical practice in order to decrease the symptom burden of patients receiving CTX and develop and test novel symptom management interventions.

## Results

### Overview of the process and results

As a first step in this study, we built on our previous research that evaluated for differences in networks created using different dimensions of the symptom experience (i.e., occurrence, severity, and distress)^13^. Using NA, we extend our previous findings by exploring how the relationships between and among symptoms and symptoms clusters change over two cycles of CTX within a large and heterogeneous sample of oncology patients with different types of cancer (i.e., breast, gastrointestinal, gynaecological, and lung), as well as within a distinct type of cancer (i.e., breast) using the occurrence dimension of the symptom experience. Considering the relationships between and among symptoms and symptom clusters, two analytic approaches can be used^14^: (1) an analysis of these relationships among symptoms in patients with various types of cancers pooled together and (2) an analysis of interconnections among symptoms within specific type of cancer (e.g., breast cancer). In this study, we evaluated the symptom clusters using both approaches and illustrate variations among the connections between and among symptoms and symptoms clusters across two cycles of CTX.

**Figure 1:**
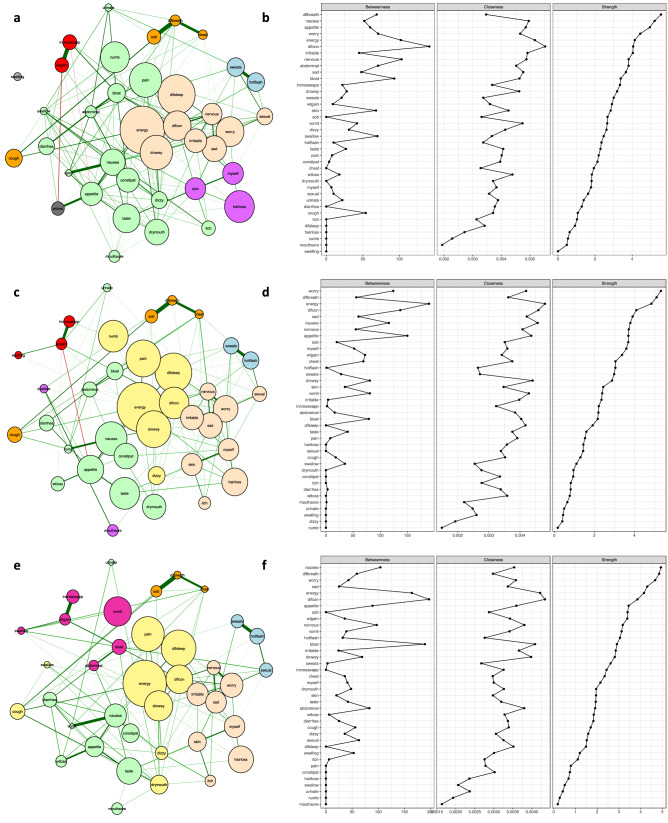
The estimated networks for 38 cancer symptoms with the identified symptom clusters and centrality indices during cycle 1 of CTX using ratings of symptom occurrence. Patients with four types of cancer (i.e., breast, gastrointestinal, gynaecological, and lung) with complete data across the six assessments (n = 987) were included in these analyses. Nodes represent symptoms and edges represent pairwise correlations between the symptoms, after conditioning on all of the other nodes in the network. Symptom clusters are depicted with different colours. Centrality indices were ordered by strength values. (**a**) Estimated network of 38 cancer symptoms with the identified clusters for time-point 1: prior to the second or third cycle of CTX administration. (**b**) Centrality indices (betweenness, closeness, and strength) for the estimated network shown in (**a**). Symptom(s) with no closeness coefficient appear separated from the rest of the network. (**c**) Estimated network for 38 cancer symptoms with the identified clusters for time-point 2: approximately 1 week after CTX administration. (**d**) Centrality indices (betweenness, closeness, and strength) for the estimated network shown in (**c**). (**e**) Estimated network for 38 cancer symptoms with the identified clusters for time-point 3: approximately 2 weeks after CTX administration. (**f**) Centrality indices (betweenness, closeness, and strength) for the estimated network shown in (**e**).

**Figure 2:**
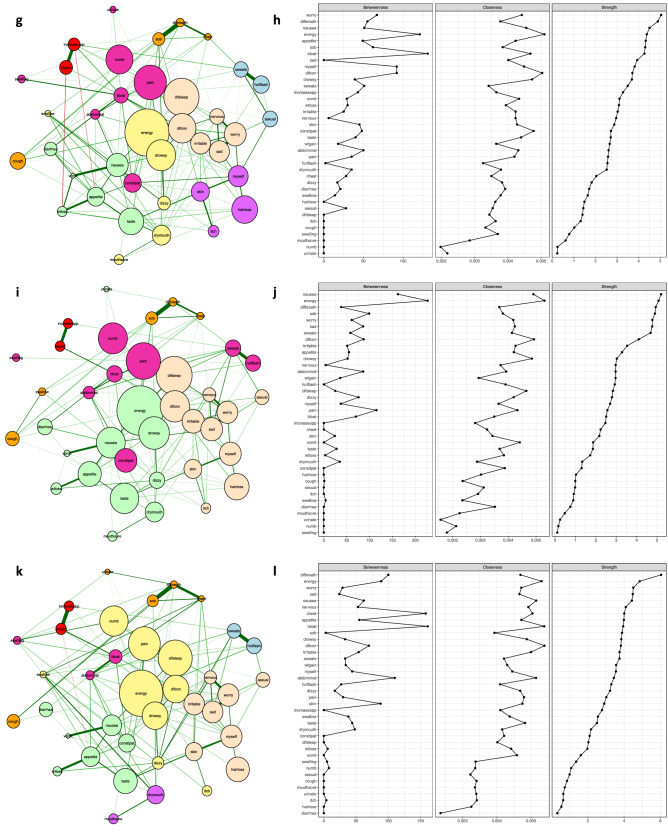
The estimated networks for 38 cancer symptoms with the identified symptom clusters and centrality indices during cycle 2 of CTX using ratings of symptom occurrence. Patients with four types of cancer (i.e., breast, gastrointestinal, gynaecological, and lung) with complete data across the six assessments (n = 987) were included in these analyses. Nodes represent symptoms and edges represent pairwise correlations between the symptoms, after conditioning on all of the other nodes in the network. Symptom clusters are depicted with different colours. Centrality indices were ordered by strength values. (**g**) Estimated network of 38 cancer symptoms with the identified clusters for time-point 4: prior to the third or fourth cycle of CTX administration. (**h**) Centrality indices (betweenness, closeness, and strength) for the estimated network shown in (**g**). (**i**) Estimated network for 38 cancer symptoms with the identified clusters for time-point 5: approximately 1 week after CTX administration. (**j**) Centrality indices (betweenness, closeness, and strength) for the estimated network shown in (**i**). (**k**) Estimated network for 38 cancer symptoms with the identified clusters for time-point 6: approximately 2 weeks after CTX administration. (**l**) Centrality indices (betweenness, closeness, strength) for the estimated network shown in (**k**).

**Figure 3:**
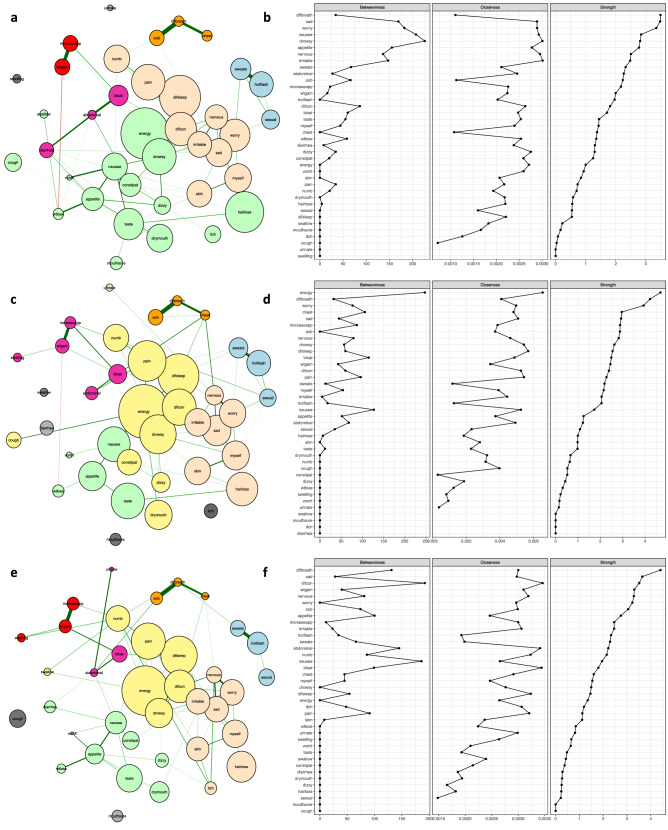
The estimated networks for 38 cancer symptoms with the identified symptom clusters and centrality indices during cycle 1 of CTX using ratings of symptom occurrence. Patients with “breast” cancer with complete data across the six assessments (n = 408) were included in these analyses. Nodes represent symptoms and edges represent pairwise correlations between the symptoms, after conditioning on all of the other nodes in the network. Symptom clusters are depicted with different colours. Centrality indices were ordered by strength values. Symptom(s) with no closeness coefficient appeared separated from the rest of the network. (**a**) Estimated network for 38 cancer symptoms with the identified clusters for time-point 1: prior to the second or third cycle of CTX administration. (**b**) Centrality indices (betweenness, closeness, and strength) for the estimated network shown in (**a**). (**c**) Estimated network for 38 cancer symptoms with the identified clusters for time-point 2: approximately 1 week after CTX administration. (**d**) Centrality indices (betweenness, closeness, and strength) for the estimated network shown in (**c**). (**e**) Estimated network for 38 cancer symptoms with the identified clusters for time-point 3: approximately 2 weeks after CTX administration. (**f**) Centrality indices (betweenness, closeness, and strength) for the estimated network shown in (**e**).

An overview of all of the data is given in the “Methods” section. The main results of this study are illustrated in Figs. 1, 2, and 3. In all of the estimated networks, the size of each node (i.e., symptom) is proportional to the occurrence rate for that symptom within each sample. Edge colours: green and red indicate positive and negative interconnections, respectively. The strength of a correlation is represented by the width and saturation of an edge. Thicker edges represent stronger associations between symptoms. The networks are considered weighted because the strength of the relationships between symptoms is taken into account. For the total sample (four types of cancer) across the six assessments (see Figs. 1 and 2) and for the patients with breast cancer (see Fig. 3) during cycle 1, undirected networks of symptoms were calculated using the IsingFit method (see “Methods”; network models of symptoms and network estimation).

In addition to finding correlations and interconnections between symptoms, the NA model was employed to determine how symptoms clustered together at various time points and for a specific type of cancer. The Walktrap algorithm (see “Methods”) was applied to identify communities/clusters of nodes/symptoms that are relatively densely connected in the network^13,15–17^. Through an iterative process, nodes were assigned into groups with small intra and larger inter-community distances using bottom-up hierarchical clustering. In Figs. 1, 2, and 3, community structures of the networks based on observations from three-six assessments over one/two cycle(s) of CTX were detected and illustrated with different colours. The number of clusters and distributions of symptoms within and between clusters were not completely identical within the various clusters, as well as across time, and within and across the samples of patients (i.e., total sample, breast cancer) that are reported in Table 1.

The centrality indices of strength, closeness, and betweenness for symptoms over time points are numerically summarised in Table S3 in the Appendix and graphically compared in Figs. 1, 2, and 3. Findings from our study provide insights as to the most important nodes/symptoms inside each network at each assessment. In addition, these measures allow for comparisons of the clinical significance of each symptom across time and across the samples that were evaluated in this study (i.e., total sample, patients with breast cancer). It should be noted that because the networks were generated using data only from patients who had complete data across all six assessments, the changes over time in which symptoms were more central in the network, were not due to differences in the number of patients whose symptoms were included in the creation of the networks. Supplementary results, including demographic information, edge-weights, centrality measures, and the stability of networks, are presented in the Appendix.

**Table 1:** Symptoms included in each of the symptom clusters derived from network analyses for the total sample and patients with breast cancer.

Symptom clusters and symptoms within the clusters	TS	TS	TS	TS	TS	TS	BC	BC	BC
	T1	T2	T3	T4	T5	T6	T1	T2	T3
	Fig. 1a	Fig. 1c	Fig. 1e	Fig. 2 g	Fig. 2i	Fig. 2k	Fig. 3a	Fig. 3c	Fig. 3e
**Psychological cluster**									
Difficulty sleeping	$\bullet$			$\bullet$	$\bullet$		$\bullet$		
Worrying	$\bullet$	$\bullet$	$\bullet$	$\bullet$	$\bullet$	$\bullet$	$\bullet$	$\bullet$	$\bullet$
Feeling sad	$\bullet$	$\bullet$	$\bullet$	$\bullet$	$\bullet$	$\bullet$	$\bullet$	$\bullet$	$\bullet$
Feeling irritable	$\bullet$	$\bullet$	$\bullet$	$\bullet$	$\bullet$	$\bullet$	$\bullet$	$\bullet$	$\bullet$
Feeling nervous	$\bullet$	$\bullet$	$\bullet$	$\bullet$	$\bullet$	$\bullet$	$\bullet$	$\bullet$	$\bullet$
Difficulty concentrating	$\bullet$			$\bullet$	$\bullet$		$\bullet$		
Lack of energy	$\bullet$								
Feeling drowsy	$\bullet$								
Problems with sexual interest or activity	$\bullet$	$\bullet$			$\bullet$	$\bullet$			
Itching		$\bullet$	$\bullet$		$\bullet$				$\bullet$
Hair loss		$\bullet$	$\bullet$		$\bullet$	$\bullet$		$\bullet$	$\bullet$
Changes in skin		$\bullet$	$\bullet$		$\bullet$	$\bullet$	$\bullet$	$\bullet$	$\bullet$
I don’t look like myself		$\bullet$	$\bullet$		$\bullet$	$\bullet$	$\bullet$	$\bullet$	$\bullet$
Pain							$\bullet$		
Numbness/tingling in hands/feet							$\bullet$		
Problems with urination								$\bullet$	
Consistency (%) 16 symptoms	56.2	56.2	50.0	37.5	68.7	50.0	62.5	50.0	50.0
**Respiratory cluster**									
Shortness of breath	$\bullet$	$\bullet$	$\bullet$	$\bullet$	$\bullet$	$\bullet$	$\bullet$	$\bullet$	$\bullet$
Difficulty breathing	$\bullet$	$\bullet$	$\bullet$	$\bullet$	$\bullet$	$\bullet$	$\bullet$	$\bullet$	$\bullet$
Cough	$\bullet$	$\bullet$		$\bullet$	$\bullet$				
Chest tightness	$\bullet$	$\bullet$	$\bullet$	$\bullet$	$\bullet$	$\bullet$	$\bullet$	$\bullet$	$\bullet$
Difficulty swallowing					$\bullet$				
Problems with urination						$\bullet$			
Consistency (%) 6 symptoms	66.7	66.7	50.0	66.7	83.3	66.7	50.0	50.0	50.0
**Gastrointestinal cluster**									
Itching	$\bullet$						$\bullet$		
Change in the way food tastes	$\bullet$	$\bullet$	$\bullet$	$\bullet$	$\bullet$	$\bullet$	$\bullet$	$\bullet$	$\bullet$
Lack of appetite	$\bullet$	$\bullet$	$\bullet$	$\bullet$	$\bullet$	$\bullet$	$\bullet$	$\bullet$	$\bullet$
Mouth sores	$\bullet$		$\bullet$		$\bullet$		$\bullet$		
Difficulty swallowing	$\bullet$						$\bullet$		
Dry mouth	$\bullet$	$\bullet$			$\bullet$		$\bullet$		$\bullet$
Vomiting	$\bullet$	$\bullet$	$\bullet$	$\bullet$	$\bullet$	$\bullet$	$\bullet$	$\bullet$	$\bullet$
Nausea	$\bullet$	$\bullet$	$\bullet$	$\bullet$	$\bullet$	$\bullet$	$\bullet$	$\bullet$	$\bullet$
Dizziness	$\bullet$				$\bullet$		$\bullet$		$\bullet$
Constipation	$\bullet$	$\bullet$	$\bullet$			$\bullet$	$\bullet$		$\bullet$
Diarrhea	$\bullet$	$\bullet$	$\bullet$	$\bullet$	$\bullet$	$\bullet$			$\bullet$
Abdominal cramps	$\bullet$	$\bullet$							
Feeling bloated	$\bullet$	$\bullet$							
Pain	$\bullet$								
Numbness/tingling in hands/feet	$\bullet$								
Problems with urination	$\bullet$	$\bullet$	$\bullet$		$\bullet$				
Weight loss		$\bullet$	$\bullet$	$\bullet$	$\bullet$	$\bullet$	$\bullet$	$\bullet$	$\bullet$
Lack of energy					$\bullet$		$\bullet$		
Feeling drowsy					$\bullet$		$\bullet$		
Cough							$\bullet$		
Hair loss							$\bullet$		
Consistency (%) 21 symptoms	76.2	52.4	42.9	28.6	57.1	33.3	71.4	23.8	42.9
**Weight gain cluster**									
Weight gain	$\bullet$	$\bullet$		$\bullet$	$\bullet$	$\bullet$	$\bullet$		$\bullet$
Increased appetite	$\bullet$	$\bullet$		$\bullet$	$\bullet$	$\bullet$	$\bullet$		$\bullet$
Swelling of the arms or legs		$\bullet$							$\bullet$
Consistency (%) 3 symptoms	66.7	100	0	66.7	66.7	66.7	66.7	0	100
**Abdominal discomfort cluster**									
Weight gain			$\bullet$					$\bullet$	
Increased appetite			$\bullet$					$\bullet$	
Abdominal cramps			$\bullet$	$\bullet$	$\bullet$	$\bullet$	$\bullet$	$\bullet$	$\bullet$
Feeling bloated			$\bullet$	$\bullet$	$\bullet$	$\bullet$	$\bullet$	$\bullet$	$\bullet$
Swelling of arm or legs			$\bullet$	$\bullet$	$\bullet$	$\bullet$		$\bullet$	
Numbness/tingling in hands/feet			$\bullet$	$\bullet$	$\bullet$				
Constipation				$\bullet$	$\bullet$				
Pain				$\bullet$	$\bullet$				
Problems with urination				$\bullet$					$\bullet$
Diarrhea							$\bullet$		
Sweats					$\bullet$				
Hot flashes					$\bullet$				
Consistency (%) 12 symptoms	0	0	50.0	58.3	66.7	25.0	25.0	41.7	25.0
**Hormonal cluster**									
Sweats	$\bullet$	$\bullet$	$\bullet$	$\bullet$		$\bullet$	$\bullet$	$\bullet$	$\bullet$
Hot flashes	$\bullet$	$\bullet$	$\bullet$	$\bullet$		$\bullet$	$\bullet$	$\bullet$	$\bullet$
Problems with sexual interest or activity			$\bullet$	$\bullet$			$\bullet$	$\bullet$	$\bullet$
Consistency (%) 3 symptoms	66.7	66.7	100	100	0	66.7	100	100	100
**Epithelial cluster**									
Hair loss	$\bullet$			$\bullet$					
Changes in skin	$\bullet$			$\bullet$					
I don’t look like myself	$\bullet$			$\bullet$					
Itching				$\bullet$					
Mouth sores		$\bullet$				$\bullet$			
Dry mouth						$\bullet$			
Difficulty swallowing		$\bullet$							
Consistency (%) 7 symptoms	42.8	28.6	0	57.1	0	28.6	0	0	0
**Sickness behaviour cluster**									
Difficulty sleeping		$\bullet$	$\bullet$			$\bullet$		$\bullet$	$\bullet$
Difficulty concentrating		$\bullet$	$\bullet$			$\bullet$		$\bullet$	$\bullet$
Lack of energy		$\bullet$	$\bullet$	$\bullet$		$\bullet$		$\bullet$	$\bullet$
Feeling drowsy		$\bullet$	$\bullet$	$\bullet$		$\bullet$		$\bullet$	$\bullet$
Dizziness		$\bullet$	$\bullet$	$\bullet$		$\bullet$		$\bullet$	
Pain		$\bullet$	$\bullet$			$\bullet$		$\bullet$	$\bullet$
Numbness/tingling in hands/feet		$\bullet$				$\bullet$		$\bullet$	$\bullet$
Cough			$\bullet$					$\bullet$	
Difficulty swallowing			$\bullet$	$\bullet$		$\bullet$			$\bullet$
Dry mouth			$\bullet$	$\bullet$				$\bullet$	
Mouth sores				$\bullet$					
Itching						$\bullet$			
Constipation								$\bullet$	
Consistency (%) 13 symptoms	0	53.8	69.2	46.1	0	69.2	0	76.9	53.8
**Single symptoms**									
Weight loss	$\bullet$								
Swelling of arms or legs	$\bullet$						$\bullet$		
Problems with urination							$\bullet$		
Itching								$\bullet$	
Mouth sores								$\bullet$	$\bullet$
Difficulty swallowing								$\bullet$	
Diarrhea								$\bullet$	
Cough									$\bullet$

The evaluation of the consistency of the symptoms was done within each cluster across the samples and across time as the total number of symptoms at a specific time point/total number of symptoms in that cluster across samples and time. Abbreviations: BC = breast cancer, T = time, and TS = total sample.

### Networks for the total sample

### Main symptom clusters

While the associations between and among symptoms and symptoms clusters over the cycles of CTX were not identified previously, in the first scenario, networks of 38 common symptoms reported by oncology patients (n = 987) with a variety of cancer types that were assessed six times over two cycles of CTX were estimated for each time-point separately. As shown in Table 1, across the samples and across time, the symptoms appear to group into six stable main clusters: psychological, respiratory, gastrointestinal, weight gain, hormonal and sickness behaviour. However, an evaluation of the consistency of the symptoms within each cluster across the samples and across time (i.e., total number of symptoms at a specific time point/total number of symptoms in that cluster across samples and time) revealed various inconsistencies.

As shown in Table 1, for the psychological cluster that was identified in all 9 NAs, the consistency of the 16 symptoms within the cluster ranged from 37.5 to 68.7%. The four symptoms that were found in all 9 NAs were worrying, feeling sad, feeling irritable, and feeling nervous. For the respiratory cluster that was identified in all 9 NAs, the consistency of the 6 symptoms within the cluster ranged from 50.0 to 83.3%. The three symptoms that were found in all 9 NAs were shortness of breath, difficulty breathing, and chest tightness. For the gastrointestinal cluster that was identified in all 9 NAs, the consistency of the 21 symptoms within the cluster ranged from 23.8 to 76.2%. The four symptoms that were found in all 9 NAs were change in the way food tastes, lack of appetite, nausea, and vomiting. For the weight gain cluster that was identified in 7 of the 9 NAs, the consistency of the 3 symptoms within the cluster ranged from 66.7 to 100.0%. The two symptoms that were found in all 7 NAs were weight gain and increased appetite. For the abdominal discomfort cluster that was identified in 7 of the 9 NAs, the consistency of the 12 symptoms within the cluster ranged from 25.0 to 66.7%. The two symptoms that were found in all 7 NAs were abdominal cramps and feeling bloated. For the hormonal cluster that was identified in 8 of the 9 NAs, the consistency of the 3 symptoms within the cluster ranged from 66.7 to 100.0%. The two symptoms that were found in all 8 NAs were sweats and hot flashes. For the epithelial cluster that was identified in 4 of the 9 NAs, the consistency of the 7 symptoms within the cluster ranged from 28.6 to 57.1%. No symptoms were found in all 4 NAs. For the sickness behaviour cluster that was identified in 6 of the 9 NAs, the consistency of the 13 symptoms within the cluster ranged from 46.1 to 76.9%. The two symptoms that were found in all 6 NAs were lack of energy and feeling drowsy.

### Network analysis for time-point 1 (i.e., prior to CTX administration for cycle 1)

Figure 1a illustrates the network of 38 common symptoms at time-point 1. Within the network, thicker edges between symptoms (e.g., difficulty breathing and shortness of breath coded as “difbreath” and “sob”, respectively in Table 2) indicate stronger associations between symptoms. In addition, increasing appetite and weight gain, sweats and hot flashes, nausea and vomiting, feeling sad and worrying are strongly correlated in pairs (Appendix Table S3). All of the connections in this network were positive except for weight gain and weight loss, and it had a medium density (i.e., 25.74% of the potential edges were connected in the network). Moreover, closer positions of symptoms in the network (e.g., lack of energy and feeling drowsy) reveal stronger/more interconnections between symptoms. Using the walktrap algorithm, the symptoms appear to group into six clusters: psychological [shown in bisque], respiratory [shown in orange], gastrointestinal [shown in green], weight gain [shown in red], hormonal [shown in blue], and epithelial [shown in purple]. To help with the interpretation of the estimated networks, the relative importance of each symptom in the network was assessed using three indices of node centrality: strength, closeness, and betweenness. Strength corresponds to the sum of the weights of the edges attached to that node. Closeness is calculated using the inverse of the sum of the distances of the node from all of the other nodes in the network. Betweenness is defined as the number of times in which a given node lies on the shortest path between two other nodes. On the subject of centrality measures, for the symptom occurrence network at time-point 1 (see Fig. 1b), difficulty concentrating had the highest scores across betweenness and closeness $({r_b}=141, {r_c}=0.005, {r_s}=4.05)$ and difficulty breathing $({r_b}=69, {r_c}=0.003, {r_s}=5.56)$, nausea $({r_b}=52, {r_c}=0.004, {r_s}=5.21)$, and lack of appetite $({r_b}=60, {r_c}=0.004, {r_s}=4.95)$ had the highest strength scores.

**Table 2:** 38 Cancer Symptoms, short codes, and symptom occurrence rates at the six time points.

Symptoms	Short codes	Occurrence rates % (n)					
		Time 1	Time 2	Time 3	Time 4	Time 5	Time 6
Lack of Energy	Energy	81.7 (806)	86.4 (853)	80.5 (795)	80.6 (796)	85.6 (845)	80.0 (790)
Difficulty Sleeping	Difsleep	68.2 (673)	69.0 (681)	64.2 (634)	64.2 (634)	66.3 (654)	62.7 (619)
Pain	Pain	59.4 (586)	64.9 (641)	60.3 (595)	59.0 (582)	63.2 (624)	58.7 (579)
Feeling Drowsy	Drowsy	59.4 (586)	64.8 (640)	52.3 (516)	53.0 (523)	56.3 (556)	47.6 (470)
Hair Loss	Hairloss	54.7 (540)	48.8 (482)	46.7 (461)	47.1 (465)	45.6 (451)	43.0 (424)
Numbness or Tingling in Hands or Feet	Numb	52.9 (522)	55.7 (550)	51.0 (503)	49.4 (488)	53.7 (530)	52.4 (517)
Worrying	Worry	51.2 (505)	46.5 (459)	42.1 (416)	39.9 (394)	38.4 (379)	38.9 (384)
Difficulty Concentrating	Difcon	50.8 (501)	55.7 (550)	49.5 (489)	50.5 (498)	54.2 (535)	48.0 (474)
Change in the Way Food Tastes	Taste	49.5 (489)	56.0 (553)	45.9 (453)	45.7 (451)	52.6 (519)	43.7 (431)
Nausea	Nausea	45.3 (488)	59.2 (585)	40.2 (397)	39.7 (392)	53.1 (525)	35.9 (355)
Feeling Sad	Sad	44.4 (438)	45.0 (444)	38.3 (378)	36.7 (362)	40.2 (397)	37.8 (373)
Dry Mouth	Drymouth	44.3 (437)	44.2 (436)	32.8 (324)	33.8 (334)	34.4 (340)	31.8 (314)
Constipation	Constipat	41.3 (408)	45.1 (445)	32.4 (320)	31.9 (315)	41.0 (405)	31.1 (307)
Feeling Irritable	Irritable	40.7 (402)	43.0 (424)	39.4 (389)	36.4 (359)	40.9 (404)	38.0 (375)
Lack of Appetite	Appetite	40.0 (395)	49.7 (491)	35.0 (345)	32.1 (317)	43.0 (424)	34.1 (337)
I Do Not Look Like Myself	Myself	38.0 (375)	38.7 (382)	38.1 (376)	36.3 (358)	40.2 (397)	39.3 (388)
Changes in Skin	Skin	37.8 (373)	39.0 (385)	33.3 (329)	32.8 (324)	32.9 (325)	31.6 (312)
Feeling Nervous	Nervous	37.0 (365)	30.3 (299)	24.6 (243)	26.8 (265)	24.0 (237)	22.9 (226)
Feeling Bloated	Bloat	32.4 (320)	30.9 (305)	26.4 (261)	26.3 (260)	28.5 (281)	23.9 (236)
Hot Flashes	Hotflash	32.0 (316)	30.5 (301)	27.4 (270)	29.9 (295)	28.5 (281)	27.7 (273)
Sweats	Sweats	32.0 (316)	28.9 (285)	23.0 (227)	26.4 (261)	28.6 (282)	25.3 (250)
Cough	Cough	31.0 (306)	27.5 (271)	27.7 (273)	28.8 (284)	25.2 (249)	23.9 (236)
Dizziness	Dizzy	29.2 (288)	31.8 (314)	23.1 (228)	23.3 (230)	28.3 (279)	21.5 (212)
Diarrhea	Diarrhea	29.2 (288)	28.0 (276)	25.8 (255)	23.3 (230)	27.7 (273)	24.9 (246)
Problems with Sexual Interest or Activity	Sexual	29.2 (288)	26.6 (263)	24.7 (244)	27.2 (268)	25.9 (256)	25.6 (253)
Increased Appetite	Increaseapp	25.7 (254)	20.7 (204)	23.5 (232)	21.9 (216)	17.4 (172)	18.1 (179)
Shortness of Breath	Sob	25.2 (249)	23.7 (234)	20.3 (200)	21.9 (216)	22.0 (217)	20.7 (204)
Weight Gain	Wtgain	25.0 (247)	20.6 (203)	20.4 (201)	23.8 (235)	18.1 (179)	19.7 (194)
Itching	Itch	25.0 (247)	22.6 (223)	19.7 (194)	20.0 (197)	18.0 (178)	17.9 (177)
Weight Loss	Wtloss	23.5 (232)	25.3 (250)	20.2 (199)	17.5 (173)	20.2 (199)	16.1 (159)
Abdominal Cramps	Abdominal	20.9 (206)	26.6 (263)	18.4 (182)	17.2 (170)	21.8 (215)	16.0 (158)
Mouth Sore	Mouthsore	20.6 (203)	20.6 (203)	20.4 (201)	17.2 (170)	17.7 (175)	17.5 (173)
Difficulty Breathing	Difbreath	19.0 (188)	17.3 (171)	13.9 (137)	14.8 (146)	14.9 (147)	13.2 (130)
Chest Tightness	Chest	16.4 (162)	15.8 (156)	11.6 (114)	11.4 (113)	12.7 (125)	10.6 (105)
Swelling	Swelling	13.9 (137)	13.3 (131)	13.2 (130)	13.8 (136)	13.7 (135)	14.3 (141)
Problems with Urination	Urinate	13.1 (129)	14.4 (142)	10.6 (105)	11.6 (114)	9.7 (96)	9.3 (92)
Difficulty Swallowing	Swallow	13.0 (128)	15.1 (149)	11.4 (113)	10.6 (105)	13.7 (135)	11.9 (117)
Vomitting	Vomit	11.3 (112)	14.2 (140)	8.4 (83)	8.4 (83)	11.1 (110)	7.9 (78)

### Network analysis for time-point 2 (i.e., approximately 1 week after CTX administration during cycle 1)

Figure 1c illustrates the relationships among symptoms at time-point 2. Symptom interactions and clusters changed compared to time-point 1. In this network, shortness of breath and difficulty breathing, hot flashes and sweats, increased appetite and weight gain, chest tightness and difficulty breathing, feeling sad and worrying were strongly correlated in pairs (Appendix Table S3). All of the relationships were positive except for lack of appetite and weight gain, and this network had a medium density (20.76%). The symptoms group into seven clusters: psychological [shown in bisque], respiratory [shown in orange], gastrointestinal [shown in green], weight gain [shown in red], hormonal [shown in blue], and epithelial [shown in purple]. In addition, a new sickness behaviour cluster [shown in yellow] was identified. Figure 1d illustrates the centrality indices for the symptom occurrence network at time-point 2. Lack of energy had the highest scores across betweenness and closeness $({r_b}=190, {r_c}=0.004, {r_s}=4.84)$ and worrying $({r_b}=124, {r_c}=0.004, {r_s}=5.32)$, difficulty breathing $({r_b}=56, {r_c}=0.003, {r_s}=5.10)$, and lack of energy had the highest values for strength.

### Network analysis for time-point 3 (i.e., approximately 2 weeks after CTX administration during cycle 1)

Figure 1e illustrates the relationship among the symptoms at time-point 3. Shortness of breath and difficulty breathing, increased appetite and weight gain, hot flashes and sweats, nausea and vomiting, and feeling sad and worrying were strongly correlated in pairs (see Appendix Table S3). All the connections in the network were positive, and it had a medium density (20.05%). Six symptom clusters were found: psychological [shown in bisque], respiratory [shown in orange], gastrointestinal [shown in green], abdominal discomfort [shown in pink] appeared as a new one, hormonal [shown in blue], and sickness behaviour [shown in yellow]. In terms of the centrality indices at time-point 3 (see Fig. 1f), difficulty concentrating has the highest scores across betweenness and closeness measures $({r_b}=197, {r_c}=0.004, {r_s}=3.84)$ and nausea $({r_b}=104, {r_c}=0.003, {r_s}=4.93)$, difficulty breathing $({r_b}=59, {r_c}=0.003, {r_s}=4.85)$, and worrying $({r_b}=43, {r_c}=0.003, {r_s}=4.66)$ had the highest strength scores.

### Network analysis for time-point 4 (i.e., prior to CTX administration during cycle 2)

Figure 2g illustrates the network of symptoms at time-point 4 with a medium density (24.32%), and all connections were positive between symptoms except for: increased appetite and lack of appetite and weight gain and weight loss. Shortness of breath and difficulty breathing, hot flashes and sweats, increased appetite and weight gain, feeling sad and worrying, and lack of energy and feeling drowsy were strongly correlated in pairs (tabulated in Appendix Table S3). The symptoms grouped into eight clusters: psychological [shown in bisque], respiratory [shown in orange], gastrointestinal [shown in green], weight gain [shown in red], abdominal discomfort [shown in pink], hormonal [shown in blue], epithelial [shown in purple], and sickness behaviour [shown in yellow]. Figure 2h illustrates the centrality indices at time-point 4. Feeling bloated has the highest betweenness score $({r_b}=131, {r_c}=0.004, {r_s}=4.26)$, lack of energy has the highest closeness score $({r_b}=121, {r_c}=0.005, {r_s}=4.37)$, and worrying $({r_b}=67, {r_c}=0.004, {r_s}=5.04)$, difficulty breathing $({r_b}=55, {r_c}=0.003, {r_s}=4.88)$, and nausea $({r_b}=51, {r_c}=0.004, {r_s}=4.50)$ had the highest strength scores.

### Network analysis for time-point 5 (i.e., approximately 1 week after CTX administration during cycle 2)

For time-point 5, see Fig. 2i, all the connections in the network were positive, and it had a medium density (21.62%). Among all of the symptoms, shortness of breath and difficulty breathing, hot flashes and sweats, increased appetite and weight gain, lack of energy and feeling drowsy, and nausea and vomiting were strongly correlated in pairs (listed in Appendix Table S3). The symptoms grouped into five clusters: psychological [shown in bisque], respiratory [shown in orange], gastrointestinal [shown in green], weight gain [shown in red], and abdominal discomfort [shown in pink]. In terms of the centrality indices at time-point 5 (see Fig. 2j), lack of energy had the highest scores across betweenness and closeness measures $({r_b}=227, {r_c}=0.005, {r_s}=5.07)$ and nausea $({r_b}=162, {r_c}=0.004, {r_s}=5.20)$, lack of energy, and difficulty breathing $({r_b}=38, {r_c}=0.003, {r_s}=4.93)$ had the highest strength scores.

### Network analysis for time-point 6 (i.e., approximately 2 weeks after CTX administration during cycle 2)

Figure 2k illustrates the network at time-point 6 with a medium density (23.61%) that displays positive connections between symptoms except for weight gain and lack of appetite. Among all of the symptoms, shortness of breath and difficulty breathing, increased appetite and weight gain, hot flashes and sweats, feeling sad and worrying, and feeling nervous and worrying were strongly correlated in pairs (listed in Appendix Table S3). The symptoms grouped into eight clusters: psychological [shown in bisque], respiratory [shown in orange], gastrointestinal [shown in green], weight gain [shown in red], abdominal discomfort [shown in pink], hormonal [shown in blue], epithelial [shown in purple], and sickness behaviour [shown in yellow]. In terms of centrality indices for time-point 6 (see Fig. 2l), feeling bloated $({r_b}=161, {r_c}=0.005, {r_s}=3.94)$ and difficulty concentrating $({r_b}=70, {r_c}=0.005, {r_s}=3.82)$ had the highest values for betweenness and closeness scores, and difficulty breathing $({r_b}=100, {r_c}=0.004, {r_s}=6.04)$, lack of energy$({r_b}=89, {r_c}=0.005, {r_s}=4.86)$, and worrying $({r_b}=29, {r_c}=0.004, {r_s}=4.50)$ had the highest strength scores.

### Networks for patients with breast cancer

To explore whether symptoms clusters and their associations were different for a specific type of cancer, we investigated the hypothesis that the interconnections between and among symptoms, as well as the stability and consistency of the clusters would be associated with a specific type of cancer. Therefore, in our second approach, separate networks were estimated for the 408 patients with breast cancer (i.e., by the type of cancer) during cycle 1 of CTX (i.e., 3 time points). For the patients with breast cancer, Fig. 3a shows the network of 38 symptoms at time-point 1. All the connections were positive except for weight gain and weight loss, and it had a low density (10.67%). Among all of the symptoms, shortness of breath and difficulty breathing, hot flashes and sweats, increased appetite and weight gain, feeling sad and worrying, and difficulty breathing and chest tightness were strongly correlated in pairs (listed in Appendix Table S3). The symptoms grouped into six clusters: psychological [shown in bisque], respiratory [shown in orange], gastrointestinal [shown in green], weight gain [shown in red], abdominal discomfort [shown in pink], and hormonal [shown in blue]. In terms of centrality indices at time-point 1, for patients with breast cancer (see Fig. 3b), feeling drowsy had the highest values for betweenness and closeness scores $({r_b}=226, {r_c}=0.003, {r_s}=2.81)$, and difficulty breathing $({r_b}=34, {r_c}=0.001, {r_s}=3.49)$, feeling sad $({r_b}=169, {r_c}=0.002, {r_s}=3.48)$, and worrying $({r_b}=182, {r_c}=0.002, {r_s}=3.35)$ had the highest strength scores.

Figure 3c illustrates the network of symptoms at time-point 2. All the connections were positive except for weight gain and weight loss, and it had a low density (11.24%). Among all of the symptoms, shortness of breath and difficulty breathing, increased appetite and weight gain, hot flashes and sweats, difficulty breathing and chest tightness, and feeling sad and worrying were strongly correlated in pairs (see Appendix Table S3).The symptoms grouped into six clusters: psychological [shown in bisque], respiratory [shown in orange], gastrointestinal [shown in green], abdominal discomfort [shown in pink], hormonal [shown in blue], and sickness behaviour [shown in yellow]. In terms of the centrality indices at time-point 2 (see Fig. 3d), lack of energy had the highest values across all three indices $({r_b}=247, {r_c}=0.005, {r_s}=4.69)$, and difficulty breathing $({r_b}=32, {r_c}=0.004, {r_s}=4.22)$ and worrying $({r_b}=77, {r_c}=0.004, {r_s}=3.93)$ were ranked second and third respectively for strength index.

Figure 3e illustrates the network of symptoms at time-point 3. All of the connections were positive, and it had a low density (10.52%). Among all of the symptoms, shortness of breath and difficulty breathing, increased appetite and weight gain, hot flashes and sweats, difficulty breathing and chest tightness, and feeling sad and worrying were strongly correlated in pairs (listed in Appendix Table S3). The symptoms grouped into seven clusters: psychological [shown in bisque], respiratory [shown in orange], gastrointestinal [shown in green], weight gain [shown in red], abdominal discomfort [shown in pink], hormonal [shown in blue], and sickness behaviour [shown in yellow]. Concerning centrality indices at time-point 3 (see Fig. 3f), difficulty concentrating had the highest betweenness and closeness scores $({r_b}=192, {r_c}=0.003, {r_s}=3.51)$, and difficulty breathing $({r_b}=131, {r_c}=0.003, {r_s}=4.41)$, feeling sad $({r_b}=28, {r_c}=0.002, {r_s}=3.65)$, and difficulty concentrating had the highest strength index.

Due to the number of patients with breast cancer and the high number of parameters that should be estimated (i.e., 38 threshold parameters and 38*37/2 = 703 pairwise association parameters) for networks with 38 nodes, we tested the stability of the estimated networks, centrality indices and identified clusters using 20 cancer symptoms (nodes). In these networks, the 20 symptoms were chosen in descending order of their occurrence rate. This approach allowed us to deal with the problem of relatively small dataset for a specific cancer type and to mitigate the bias in the network construction by repeating the experiments several times and with a sub-sample of the data. Considering the removed nodes, we obtained similar results for the node strength for the new estimated networks. This new set of experiments with 20 cancer symptoms and associated results are found in the supplementary document (Fig. S6).

### Summary of findings

Taken together, these findings suggest that, when networks are created separately for patients with a specific type of cancer (i.e., breast), the estimated networks and some of the derived symptoms clusters, as well as the interconnections among symptoms, are dependent on the type of cancer. For example, during cycle 1 of CTX at time-point 1 for the patients with breast cancer, the respiratory and psychological clusters warrant additional evaluation based on strength scores. However, for the total sample, the symptoms within the respiratory and gastrointestinal clusters need to be addressed. Furthermore, an evaluation of the stability of the symptom clusters, as well as the strength score, over 1 or 2 cycles of CTX, suggest that the psychological cluster and its consistent symptoms were relatively stable across time among patients with breast cancer. On the contrary, for the total sample, in the early and late stages of CTX (time-point 2 during cycle 1, and time-point 6 during cycle 2) the psychological cluster warrants careful assessment and appropriate referrals for counseling.

In sum, results for both the total sample and within a type of cancer suggest that changes occur over time in the relationships and interconnections between and among symptoms and symptoms clusters when one evaluates these networks over one to two cycle(s) of CTX. As shown in Table 1, across these analyses, eight unique symptom clusters were identified. Within each cluster, the specific symptoms and number of symptoms varied across time when the networks were created using the total sample and for a type of cancer. However, within each cluster, across all of the networks that were estimated (i.e., 9 in total), core or sentinel symptoms were identified. Through the use of NA, knowledge of the structural relationships between and among symptoms and symptoms clusters is increased. This information will inform future research that aims to identify core or sentinel symptoms that are driving various associations within and across symptom networks over time.

### Accuracy and stability of the networks

Network structures are inherently sensitive to the specific data and variables included in a study and to the specific estimation methods that are employed. This sensitivity poses a major challenge, especially when substantive interpretations such as treatment recommendations or the generalisability of the findings are important. The accuracy of all of the estimated network models was analysed using the non-parametric bootstrapping technique^18^ that creates new plausible datasets from resampling of the original data. This procedure was performed to compute 95% confidence intervals (CI) for the estimated edge weights accuracy for each network. Large overlapping bootstrapped CIs warrant caution when interpreting edges in the network. In addition, to assess the stability of centrality indices, case-dropping and node-dropping sub-setting bootstrap methods with the correlation stability (Cs) coefficient, that is a measure used to quantify the stability of the centrality indices, were applied. Bootstrapped CIs around the estimated edge-weights, indicate that all networks were estimated with small to moderate CIs (shown in Appendix Fig. S1). Results illustrated a degree of overlapping among the edges’ 95% CI. The results of the case-dropping and node-dropping bootstrap techniques that were used to estimate the stability of the centrality indices are shown in Appendix Figs. S2 and S3. For the robustness analysis, the CS-coefficients of the bootstrap subsets of each network are listed in Table 3. The value for the CS-coefficients for all of the networks indicates that strength was the moderately more stable centrality index than betweenness and closeness.Therefore, the order of node strength can be interpreted for symptom management strategies, while the betweenness and closeness can not. In addition, we tested the stability of the centrality indices on three equally divided and randomly assigned subsets (Fig. S4). Similar results were obtained for the node strength for each symptom network across time points (Fig. S5) for this scenario. These analyses showed the stability of the identified networks as well as the repeatability of the NA approach on cancer symptoms.

**Table 3:** Correlation stability (CS) coefficients for all networks.

Networks	CS-coefficients		
	Betweenness	Closeness	Strength
All patients at time-point 1 (Fig. 1a)	0.05	0.05	0.59
All patients at time-point 2 (Fig. 1c)	0.2	0.05	0.67
All patients at time-point 3 (Fig. 1e)	0.13	0.05	0.75
All patients at time-point 4 (Fig. 2g)	0.05	0.05	0.52
All patients at time-point 5 (Fig. 2i)	0.05	0.05	0.67
All patients at time-point 6 (Fig. 2k)	0.13	0.05	0.59
Patients with breast cancer at time-point 1 (Fig. 3a)	0.13	0.05	0.52
Patients with breast cancer at time-point 2 (Fig. 3c)	0.13	0.05	0.36
Patients with breast cancer at time-point 3 (Fig. 3e)	0.05	0.05	0.67

## Discussion

This study is the first to use NA methods to identify symptom clusters, as well as changes over time in the inter-relationships between and among 38 symptoms and symptom clusters in a large sample of oncology patients who were assessed over two cycles of CTX. The findings from this study demonstrate the unique advantages of using NA methods to examine these relationships over other methods like cluster analysis and EFA^19^. The analyses in this paper are noteworthy because we evaluated changes over time in symptom clusters in a large sample that was heterogeneous in terms of their types of cancer, as well as in patients with a very common type of cancer, namely breast. Equally important, an evaluation of the centrality measures provides new insights into changes in core/sentinel symptoms within the various networks that suggest targets for therapeutic interventions^20–24^.

**Figure 4:**
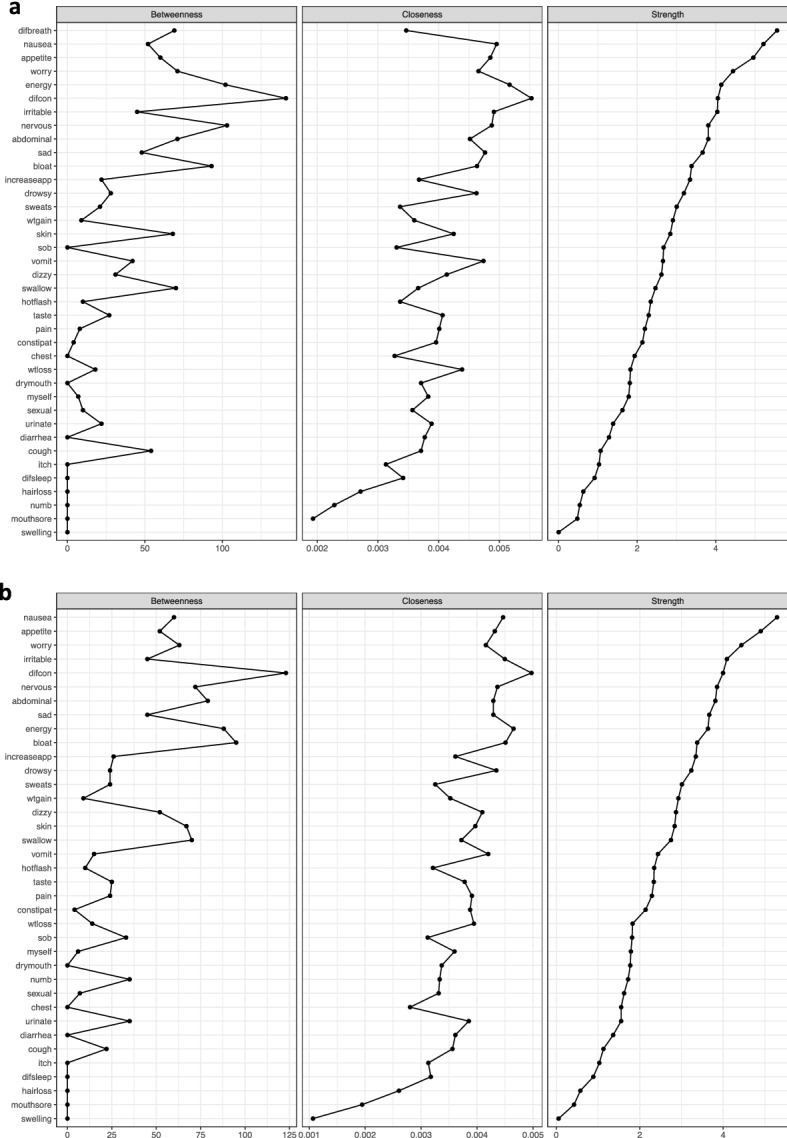
Comparison of centrality indices (betweenness, closeness, strength). Centrality indices ordered by strength values. Symptom(s) with no closeness coefficient appeared separated from the rest of the network. (**a**) Centrality indices (betweenness, closeness, and strength) for the estimated network shown in Fig. 1a. (**b**) Centrality indices (betweenness, closeness, and strength) for the estimated network at time-point 1 after removing difficulty breathing node.

Specifically, the centrality indices provide new information about the structural importance of individual symptoms within each network. As we noted in the results section for the total sample (see Figs. 1 and 2) and considering the stability of centrality indices (see Table 3), among the 38 symptoms evaluated, difficulty breathing, nausea, and lack of appetite had the highest ranks across the strength scores at time-point 1. These three symptoms which are representative of respiratory and gastrointestinal clusters may be targets for tailored interventions. Given that, worrying, difficulty breathing, and lack of energy symptoms had the highest strength scores among all symptoms at time-point 2, they may warrant intervention in the week following CTX administration. Referring to Table 1, an intervention to decrease worrying may result in a reduction in the other symptoms within the psychological cluster (i.e., feeling sad, feeling irritable, feeling nervous, problems with sexual interest or activity, itching, hair loss, changes in skin, and I don’t look like myself). Moreover, an exercise intervention to decrease fatigue (i.e., lack of energy) may diminish other symptoms in the sickness behaviour cluster (e.g., difficulty sleeping, difficulty concentrating, feeling drowsy, dizziness, pain, and numbness/tingling in hands/feet). Similarly, symptoms within the gastrointestinal, respiratory, and psychological clusters at time-point 3 may have higher priorities for clinical interventions. At time-points 4 and 5, symptoms within the sickness behaviour, respiratory, and gastrointestinal clusters may warrant tailored treatments during these phases of CTX. At time-point 6, symptoms within the respiratory, sickness behaviour, and psychological clusters may warrant additional assessments and treatments. For patients with breast cancer (see Fig. 3), at time-point 1, our findings suggest that respiratory and psychological clusters warrant clinical evaluation and management. At time-points 2 and 3, sickness behaviour, respiratory, and psychological clusters may warrant comprehensive assessments and tailored interventions. While the directionality of these effects is not clear, it is possible that intervening on a single symptom or multiple symptoms withing a cluster, anywhere in the CTX cycle, may have a positive effect on other symptoms and lead to better long-term outcomes. To test this hypothesis, while only 19.0% of the patients in the total sample reported difficulty breathing prior to their next dose of CTX at time-point 1, when the difficulty breathing node (symptom) was removed from the network, as shown in Fig. 4, it changed the centrality measures for the rest of the symptoms in the network. Therefore, if difficulty breathing can be diminished, the other correlated symptoms/nodes within the symptom cluster may decrease as well. Future analyses that remove core/sentinel symptoms from a network based on their centrality measures are required to evaluate this hypothesis. These types of analyses are particularly useful for those sentinel symptoms that have effective interventions available for them. If the NA revealed that the removal of a symptom from the network resulted in favourable changes in the network and an effective intervention was available for that sentinel symptom, one could develop an intervention study to evaluate this hypothesis.

While a large amount of research has evaluated symptom clusters in oncology patients^3,25^, our study similar to^13,26^ used NA to visualise how one symptom cluster is associated with other symptom clusters. Cluster analysis and EFA techniques are the most common approaches that are used to evaluate symptom clusters^8,9,26–29^. While these methods have identified a number of common symptom clusters in oncology patients (e.g., psychological cluster and gastrointestinal cluster), these symptom clusters are created independently; do not allow for an evaluation of the strength of the interactions among symptoms within a cluster; and do not support an examination of the relationships between and among the various symptom clusters. Our method represents a breakthrough in symptom cluster research. Within each network, our graphical representation allows us to visualise how the various symptoms and symptom clusters within the network are interconnected and how these networks change over time and within and across different samples of oncology patients.

Central questions in symptom cluster research include whether symptom clusters are stable over time and across cancer types. An evaluation of the information summarised in Table 1 provides some insights into these questions. As reported in our previous publications that used EFA to identify symptom clusters^7–9^, we suggested that within patients with breast, lung, and gastrointestinal cancers, symptom clusters remained relatively stable over time. Of note, in our NA, the three most common symptom clusters identified across studies of oncology patients were psychological, respiratory, and gastrointestinal clusters in the total sample and in patients with breast cancer at every time point. NA allowed us to discover a core set of symptoms and their interconnections that were associated with each symptom cluster. For instance, for the psychological cluster, a core set of symptoms were worrying, feeling sad, feeling irritable, and feeling nervous. If these findings are replicated, they can be used by clinicians to guide their assessments of oncology patients’ psychological status by using a specific set of core symptoms.

In conclusion, we used NA to discover the inter-relationships among 38 common symptoms in oncology patients over two cycles of CTX. Our work provides new insights into the interconnections among symptoms and symptom clusters over time. Findings from this research suggest that while the inter-relationships between and among symptoms change, they are relatively stable over time for a number of symptoms within each cluster. The use of NA has the potential to improve our understanding of oncology patients’ symptom experiences; to identify core or sentinel symptoms within a network; specify the significance of changes in these core/sentinel symptoms over time; and determine in a clinical trial that intervenes on a core symptom in the network if the interventions decrease the symptom burden of oncology patients.

## Limitations and future directions

The heterogeneity among patients receiving chemotherapy in this study (e.g. demographic, disease stage, treatments and clinical characteristics) may influence the stability of the estimated networks. For future research, larger sample sizes are needed to be able to evaluate, using NA, for differences in symptom clusters based on differences in various demographic characteristics (e.g., age, gender), types of cancer, stages of disease, and types of cancer treatment (e.g. surgery, radiation). Comparisons of network structures based on these aforementioned characteristics may assist clinicians to provide more targeted and personalised interventions.

## Methods

### Dataset

This study is part of a descriptive, longitudinal study that evaluated the symptom experience of oncology outpatients receiving CTX. The methods for this study are described in detail elsewhere^2,30,31^. Patients were recruited during their first or second cycle of CTX and were followed for two additional cycles. Six assessments were done over 2 cycles of CTX: time-point 1- prior to CTX administration, time-point 2- approximately 1 week after CTX administration, time-point 3- approximately 2 weeks after CTX administration for cycle 1 (i.e., second or third dose of CTX), and time-point 4- prior to CTX administration, time-point 5- approximately 1 week after CTX administration, and time-point 6- approximately 2 weeks after CTX administration for cycle 2 (i.e., third or fourth dose of CTX).

Patients were eligible to participate if they: were $\ge$ 18 years of age; had a diagnosis of breast, gastrointestinal, gynecological, or lung cancer; had received CTX within the preceding four weeks; were scheduled to receive at least two additional cycles of CTX; were able to read, write, and understand English; and gave written informed consent. Patients were recruited from two Comprehensive Cancer Centers, one Veteran’s Affairs hospital, and four community-based oncology programs. This study was approved by the Committee on Human Research at the University of California, San Francisco and at each of the study sites. All methods were performed in accordance with the relevant guidelines and regulations. Written informed consent was obtained from all patients.

To assess the patient’s symptom experience,^32–35^, a modified version of the Memorial Symptom Assessment Scale (MSAS) was used to evaluate the occurrence, severity, frequency, and distress of 38 symptoms commonly associated with cancer and its treatment. A description of the 38 common cancer symptoms, short codes (node labels) utilised in the NA, and symptoms occurrence rates at the six time points are listed in Table 2. Among 1328 participants registered for this study, a total of 987 patients provided complete data for all six assessments. Of the total sample $(n=987)$, 78.9% were female. Among the types of cancer, 41.3% of the patients had breast, 29.8% had gastrointestinal, 17.7% had gynaecological, and 11.2% had lung cancer. Additional demographic and clinical characteristics of the total sample are summarised in Table 4. Characteristics of the patients with breast cancer are provided in Table S1 in the Appendix. Data from the occurrence dimension of the symptom experience (i.e., whether or not the patient experienced each symptom in the past week) were used in the NA to examine the interconnections between co-occurring symptoms and symptom clusters. Separate networks were created for each of the six time points for the total sample, as well as for patients with breast cancer.

**Table 4:** Demographic and clinical characteristics of the total sample (n = 987). Abbreviations: CTX = chemotherapy, RT = radiation therapy, SD = standard deviation.

Characteristic	Mean (SD)
Age (years)	56.9 (12.0)
Education (years)	16.2 (3.0)
Body mass index (kilograms/meter$$^{2}$$)	26.3 (5.8)
Karnofsky performance status score	80.7 (12.2)
Number of comorbidities	2.4 (1.4)
Self-administered Comorbidity Questionnaire score	5.4 (3.2)
Alcohol Use Disorders Identification Test score	3.0 (2.6)
Time since cancer diagnosis (years)	1.9 (3.8)
Time since cancer diagnosis (median)	0.42
Number of prior cancer treatments	1.6 (1.5)
Number of metastatic sites including lymph node involvement	1.2 (1.2)
Number of metastatic sites excluding lymph node involvement	0.8 (1.1)
	**% (n)**
Female (% yes)	78.9 (779)
**Ethnicity**	
White	70.3 (685)
Black	6.8 (66)
Asian or Pacific Islander	12.6 (123)
Hispanic Mixed or Other	10.4 (101)
Married or partnered (% yes)	65.1 (663)
Lives alone (% yes)	22.0 (214)
Child care responsibilities (% yes)	22.2 (215)
Care of adult responsibilities (% yes)	7.9 (71)
Currently employed (% yes)	34.4 (336)
**Income**	
$${<}$$ $30,000$$+$$	17.1 (150)
$30,000 to <$70,000	20.9 (183)
$70,000 to <$100,000	17.1 (150)
$$\geqslant $$$100,000	44.8 (392)
**Specific comorbidities (% yes)**	
Heart disease	4.7 (46)
High blood pressure	30.1 (297)
Lung disease	9.8 (97)
Diabetes	8.6 (85)
Ulcer or stomach disease	4.9 (48)
Kidney disease	1.6 (16)
Liver disease	6.0 (59)
Anemia or blood disease	12.4 (122)
Depression	18.6 (184)
Osteoarthritis	11.6 (114)
Back pain	25.0 (247)
Rheumatoid arthritis	3.5 (35)
Exercise on a regular basis (% yes)	71.7 (693)
Smoking, current or history of (% yes)	34.6 (337)
**Cancer diagnosis**	
Breast	41.3 (408)
Gastrointestinal	29.8 (294)
Gynecological	17.7 (175)
Lung	11.2 (110)
**Type of prior cancer treatment**	
No prior treatment	24.2 (234)
Only surgery, CTX, or RT	42.7 (412)
Surgery & CTX, or Surgery & RT, or CTX & RT	20.3 (196)
Surgery & CTX & RT	12.7 (123)

### Network models of symptoms

Networks are studied in many different scientific disciplines from biology^36,37^ and physics^38^ to the psychology^39,40^ and social sciences^41^. Network models use graphs to describe statistical relationships between phenomena^42^. In terms of discovering associations and interconnections among symptoms^20,39,43–45^ NA has been used in both psychiatry^13,46^ and oncology^47,48^. Networks are composed of two elements: a set of nodes (units) and a set of edges (connections). The edges in a network represent conditional dependencies. If an edge exists between symptom A and B, they are related even after conditioning on all of the other variables in the network^49^. For example, in symptom assessment, nodes in a network involve symptoms that patients are experiencing, whereas edges (connections) indicate to what extent symptoms are interconnected. Depending on the type of network computational model utilised, edges can be undirected, indicating mutual connection without directionality between nodes, or directed, indicating that one element has a directional predictive influence on another. Moreover, edges can be weighted or unweighted or can be positive or negative, depending on whether the activation of one node is directly or inversely proportional to the manifestation of the other node. The most popular method is to estimate undirected network models, indicating pairwise interactions called Pairwise Markov Random Fields (PMRF)^50–52^, has been used extensively to model complex behaviours in physical systems. A PMRF is a network in which nodes represent variables, connected by undirected edges (edges with no arrowhead) that indicate conditional dependence between two variables. Two variables that are not connected are independent after conditioning on other variables. The occurrence dimension of the symptom experience represents information regarding the presence or absence of a symptom. Therefore, this dimension encompasses binary data. When the data are binary, the appropriate PRMF model to use is called the Ising model^53^. The Ising model is the binary equivalent of the Gaussian approximation method by regression approach which is described in more detail in the “Networks estimation” section. To create the networks, we utilised the generalisation of the Ising model presented in the IsingFit R-package^53^. The number of parameters in PMRF network estimation thoroughly depends on the size of the network^18^. Therefore, in the case of exclusive networks based on the number of nodes and number of patients/observations, we only developed networks for the symptoms reported by patients with breast cancer. For all network estimations, the EBIC gamma hyperparameter was set to 0.25. The Ising model estimations for all of the networks used the same parameters and the OR rule for the nodewise estimation. The layout of these networks is based on the Fruchterman-Reingold algorithm, that estimates the optimal layout so that nodes with stronger and/or more connections are placed closer to each other^54^.

### Network estimation

The model used in the IsingFit^53^ is a binary equivalent of the Gaussian approximation method and is based on the Ising model^55^. In this model, variables can be in either of two states, and interactions are considered pairwise. The aforementioned model contains two node-specific parameters: the pairwise interaction parameter ${\beta _{jk}}$, representing the interaction strength between variable *j* and *k*, and the node parameter ${\tau _j}$, can be interpreted as the tendency of the variable to take the value 1, regardless of its neighbours. The IsingFit model estimates the required parameters using logistic regression. Iteratively, every variable is regressed on all of the other variables. However, to obtain sparsity, an ${l_1}$-penalty is imposed on the regression coefficients. This technique is commonly called the lasso (least absolute shrinkage and selection operator) and optimises neighbourhood selection in a computationally efficient way. The level of shrinkage depends on the penalty parameter of the lasso. In the IsingFit method, the Extended Bayesian Information Criterion (EBIC) is used to select the set of neighbour nodes that yield the lowest EBIC and in this way generates the final network. By viewing ${X_j}$ as the response variable and all the other variables ${X_{\backslash _j}}$ as the predictors, the EBIC is represented as:

1 $$\begin{aligned} BIC_\gamma (j)= & {} -2l(\hat{\Theta })+|J|\cdot \log (n)+2\gamma |J|\cdot \log (p-1), \end{aligned}$$

2 $$\begin{aligned} l(\hat{\Theta })= & {} \sum _{i=1}^{n}\Bigg ({\tau _j}{x_{ij}}+\sum _{k\in {V_{\backslash _j}}}{\beta _{jk}}{x_{ij}}{x_{ik}} - \log \Bigg (1+\exp \Bigg \{{\tau _j}+\sum _{k\in {V_{\backslash _j}}}{x_{ik}}{\beta _{jk}}\Bigg \}\Bigg )\Bigg ) \end{aligned}$$

in which ${l(\hat{\Theta })}$ is the log likelihood of the conditional probability of ${X_j}$ given its neighbours ${X_{ne(j)}}$ over all observations, |*J*| is the number of neighbours selected by logistic regression at a certain penalty parameter ${\rho }$, *n* is the number of observations, ${p-1}$ is the number of covariates (predictors), and ${\gamma }$ is a hyperparameter, determining the strength of prior information on the size of the model space. Eventually, the model with the set of neighbours that has the lowest EBIC is selected.

### Walktrap algorithm

Walktrap proposed by Pons and Latapy^17^ is a robust algorithm in graph theory used to identify communities/clusters in the networks via random walks regardless of network size^16,56^. Moreover, random walks have been studied for many decades in networks with a variety of structures to extract information about important nodes or dense groups of nodes in a network^57^. The Walktrap algorithm is a hierarchical clustering algorithm based on the principle that random walks on a network tend to get trapped into densely connected parts referring to the communities. In this method, the authors^17^ propose using a node similarity measure based on short walks to capture structural similarities between nodes to identify communities via hierarchical agglomeration. Starting by assigning each node to its own community, the distances between all adjacent nodes are computed. Then, according to the minimum of their distances, two adjacent communities are chosen, they are merged into a new one, and the distances between communities are updated. This step is repeated ${N-1}$ times, by minimising the overall distance between nodes and communities. The algorithm eventually returns a hierarchical structure of communities called a dendrogram and the algorithm stops when a partition with maximum modularity is obtained. Walktrap is preferred when researchers want flexibility in choosing community structures or the ability to explore several different cut points for communities since Walktrap returns a dendrogram^56^.

### Networks assessment

The relative importance of each node (i.e., symptom) for the overall network configuration is evaluated by its centrality^58,59^. Different methods exist to calculate a node’s centrality. Each method is based on different assumptions of network information flow^60^. Overall centrality is assessed by interpreting these measures in combination. Among the most commonly used centrality measures are: strength (sum of the weights of connected edges), closeness (inverse of the sum of the distances of a node from all other), and betweenness (sum of times in which a given node bridges the shortest path between two other nodes). Therefore, to quantify the importance of each symptom in the network, these three indices of node centrality were calculated for each of the networks.

### Networks accuracy and stability

The accuracy of the estimated network is analysed by calculating the 95% confidence intervals (CI) of the edge weight values using bootstrapping. To investigate the stability of centrality indices, a case/node dropping bootstrap is performed by dropping various proportions of cases/nodes from the network in order to observe the correlation between original centrality indices and those generated from subsets with dropped cases/nodes. A CS-coefficient estimates the maximum number of cases that can be dropped from the original sample to retain a correlation of 0.7 or greater (ranging from 0–1; values above 0.25 imply moderate stability, above 0.5 strong stability) with 95% probability between the original network and the networks with a subset of cases. CS-coefficients were calculated for three measures of node centrality: betweenness, closeness, and strength for each of the networks. For all analyses, 1000 bootstrap iterations were performed. These methods are described in detail in^18^.

## Supplementary Information

Supplementary Information.

## Data Availability

The data used in this study is available upon reasonable request and subject to ethics approval. All data requests should be sent to Dr. Christine Miaskowski (chris.miaskowski@ucsf.edu).

## Code availability

The code used in this study is available upon request to the first author, Elaheh Kalantari (e.kalantari@surrey.ac.uk).
