# Supplementary material for: Network analysis to identify symptoms clusters and temporal interconnections in oncology patients
Source: Sci Rep. 2022 Oct 12;12:17052. doi: 10.1038/s41598-022-21140-4 (PMC9556713; doi:10.1038/s41598-022-21140-4)
Supplement: Supplementary file 1 — Supplementary Information. [file 41598_2022_21140_MOESM1_ESM.pdf]

Supplementary Information for “Network Analysis to Identify Symptoms Clusters and Temporal Interconnections in Oncology Patients”

Elaheh Kalantari[1,4 *], Samaneh Kouchaki[1,4], Christine Miaskowski[2 *], Kord Kober[2], Payam Barnaghi[3 *,4]

[1]Centre for Vision, Speech and Signal Processing (CVSSP), University of Surrey, UK.
[2]Department of Physiological Nursing, University of California San Francisco, USA.
[3]Department of Brain Sciences, Imperial College London, UK.
[4]UK Dementia Research Institute Care Research and Technology Centre, Imperial College London and the University of Surrey, UK.

Supplemental Tables S1-S2-S3

Table S1 | Demographic and clinical characteristics of the patients with breast cancer (n=408). Abbreviations: CTX = chemotherapy, RT = radiation therapy, SD = standard deviation.

**Characteristic**	**Mean (SD)**
Age (years)	53.5 (11.6)
Education (years)	16.5 (3.0)
Body mass index (kilograms/meter^2)	26.3 (5.8)
Karnofsky Performance Status score	81.2 (11.5)
Number of comorbidities	2.2 (1.3)
Self-administered Comorbidity Questionnaire score	5.0 (2.8)
Alcohol Use Disorders Identification Test score	2.8 (2.4)
Time since cancer diagnosis (years)	2.5 (4.7)
Time since cancer diagnosis (median)	0.42
Number of prior cancer treatments	1.7 (1.8)
Number of metastatic sites including lymph node involvement	0.9 (1.2)
Number of metastatic sites excluding lymph node involvement	0.5 (1.0)
	**% (n)**
Female (% yes)	99.3 (405)
Ethnicity	
White	68.8 (278)
Black	5.4 (22)
Asian or Pacific Islander	14.6 (59)
Hispanic Mixed or Other	11.1 (45)
Married or partnered (% yes)	67.9 (273)
Lives alone (% yes)	17.4 (70)
Child care responsibilities (% yes)	30.0 (119)
Care of adult responsibilities (% yes)	8.1 (30)
Currently employed (% yes)	39.8 (161)
Income	
<$30,000+	13.9 (50)
$30,000 to <$70,000	17.5 (63)
$70,000 to <$100,000	16.9 (61)
⩾$100,000	51.7 (186)
Specific comorbidities (% yes)	
Heart disease	3.9 (16)
High blood pressure	22.8 (297)
Lung disease	3.9 (16)
Diabetes	6.9 (28)
Ulcer or stomach disease	3.2 (13)
Kidney disease	1.2 (5)
Liver disease	3.9 (16)
Anemia or blood disease	13.2 (54)
Depression	22.1 (90)
Osteoarthritis	10.8 (44)
Back pain	25.2 (103)
Rheumatoid arthritis	3.2 (13)
Exercise on a regular basis (% yes)	76.2 (301)
Smoking, current or history of (% yes)	29.5 (120)
Type of prior cancer treatment	
No prior treatment	27.5 (110)
Only surgery, CTX, or RT	42.0 (168)
Surgery & CTX, or Surgery & RT, or CTX & RT	14.2 (57)
Surgery & CTX & RT	16.3 (65)

Table S2 | Summary of edge-weights for symptoms of oncology patients over time

Symptom 1	Symptom 2	Rank	edge-weight
All patients at time-point 1			
Difficulty Breathing	Shortness of Breath	1	2.53
Increased Appetite	Weight Gain	2	2.39
Sweats	Hot Flashes	3	1.88
Nausea	Vomitting	4	1.65
Feeling Sad	Worrying	5	1.6
All patients at time-point 2			
Difficulty Breathing	Shortness of Breath	1	3.02
Sweats	Hot Flashes	2	2.22
Increased Appetite	Weight Gain	3	2.1
Chest Tightness	Difficulty Breathing	4	1.88
Feeling Sad	Worrying	5	1.61
All patients at time-point 3			
Difficulty Breathing	Shortness of Breath	1	2.97
Increased Appetite	Weight Gain	2	2.43
Sweats	Hot Flashes	3	2.33
Nausea	Vomitting	4	2.05
Feeling Sad	Worrying	5	1.64
All patients at time-point 4			
Difficulty Breathing	Shortness of Breath	1	2.90
Sweats	Hot Flashes	2	2.23
Increased Appetite	Weight Gain	3	2.17
Feeling Sad	Worrying	4	1.72
Lack of Energy	Feeling Drowsy	5	1.57
All patients at time-point 5			
Difficulty Breathing	Shortness of Breath	1	3.39
Sweats	Hot Flashes	2	2.52
Increased Appetite	Weight Gain	3	2.34
Lack of Energy	Feeling Drowsy	4	1.65
Nausea	Vomitting	5	1.59
All patients at time-point 6			
Difficulty Breathing	Shortness of Breath	1	2.8
Increased Appetite	Weight Gain	2	2.48
Sweats	Hot Flashes	3	2.42
Feeling Sad	Worrying	4	1.65
Feeling Nervous	Worrying	5	1.54
Patients with breast cancer at time-point 1			
Difficulty Breathing	Shortness of Breath	1	2.11
Sweats	Hot Flashes	2	1.83
Increased Appetite	Weight Gain	3	1.73
Feeling Sad	Worrying	4	1.61
Chest Tightness	Difficulty Breathing	5	1.38
Patients with breast cancer at time-point 2			
Difficulty Breathing	Shortness of Breath	1	2.75
Increased Appetite	Weight Gain	2	1.91
Sweats	Hot Flashes	3	1.74
Chest Tightness	Difficulty Breathing	4	1.47
Feeling Sad	Worrying	5	1.38
Patients with breast cancer at time-point 3			
Difficulty Breathing	Shortness of Breath	1	2.70
Increased Appetite	Weight Gain	2	2.25
Sweats	Hot Flashes	3	2.11
Chest Tightness	Difficulty Breathing	4	1.37
Feeling Sad	Worrying	5	1.22

Table S3 | Summary of centrality indices (standardised scores) for symptoms of oncology patients over time

Symptom	Betweenness	Rank	Closeness	Rank	Strength	Rank
All patients at time-point 1						
Difficulty Concentrating	2.93	1	1.97	1	1.06	
Nervous	1.88	2	1.14	5	0.88	
Lack of Energy	1.85	3	1.51	2	1.12	5
Feeling Bloated	1.61	4	0.84		0.57	
Worrying	1.00	5	0.87		1.34	4
Nausea	0.48		1.24	3	1.89	2
Feeling Irritable	0.29		1.19	4	1.05	
Difficulty Breathing	0.95		-0.62		2.14	1
Lack of Appetite	0.70		1.11		1.71	3
All patients at time-point 2						
Lack of Energy	3.00	1	1.67	1	1.77	3
Lack of Appetite	2.19	2	1.11	5	0.97	
Difficulty Concentrating	1.93	3	1.40	2	1.25	4
Worrying	1.67	4	0.90		2.11	1
Nausea	1.51	5	1.37	3	1.04	
Feeling Drowsy	0.81		1.17	4	0.40	
Difficulty Breathing	0.31		0.16		1.96	2
Feeling Sad	0.39		0.92		1.12	5
All patients at time-point 3						
Difficulty Concentrating	2.99	1	1.95	1	1.16	
Feeling Bloated	2.83	2	1.51	3	0.44	
Lack of Energy	2.34	3	1.73	2	1.39	5
Nausea	1.16	4	0.60		1.98	1
Nervous	1.03	5	1.05	5	0.65	
Feeling Drowsy	0.48		1.34	4	0.37	
Difficulty Breathing	0.28		-0.32		1.92	2
Worrying	-0.02		0.68		1.78	3
Feeling Sad	-0.37		0.32		1.50	4
All patients at time-point 4						
Feeling Bloated	2.88	1	1.14	5	1.19	
Lack of Energy	2.58	2	1.77	1	1.27	4
I Do Not Look Like Myself	1.71	3	0.86		0.84	
Difficulty Concentrating	1.71	4	1.67	2	0.76	
Worrying	0.96	5	0.75		1.78	1
Constipation	0.39		1.28	3	0.00	
Feeling Drowsy	0.12		1.21	4	0.75	
Difficulty Breathing	0.60		-0.20		1.67	2
Nausea	0.48		0.96		1.37	3
Lack of Appetite	0.42		0.48		1.24	5
All patients at time-point 5						
Lack of Energy	3.67	1	1.89	1	1.69	2
Nausea	2.39	2	1.45	3	1.77	1
Pain	1.47	3	0.81		0.04	
Shortness of Breath	1.15	4	0.23		1.57	4
Difficulty Concentrating	0.92	5	1.47	2	1.05	
Feeling Drowsy	0.23		1.39	4	0.34	
Difficulty Sleeping	-0.29		1.17	5	0.22	
Difficulty Breathing	-0.03		0.09		1.59	3
Worrying	0.41		0.65		1.48	5
All patients at time-point 6						
Feeling Bloated	2.90	1	1.46	2	0.78	
Chest Tightness	2.83	2	0.99		0.82	
Abdominal Cramps	1.70	3	1.13	5	0.42	
Difficulty Breathing	1.46	4	0.51		2.23	1
Continued on next page						

continued from previous page						
**Symptom**	**Betweenness**	**Rank**	**Closeness**	**Rank**	**Strength**	**Rank**
Lack of Energy	1.20	5	1.36	3	1.41	2
Difficulty Concentrating	0.76		1.47	1	0.69	
Nausea	0.57		1.14	4	1.11	5
Worrying	-0.20		0.54		1.16	3
Feeling Sad	-0.31		0.47		1.14	4
Patients with breast cancer at time-point 1						
Feeling Drowsy	2.67	1	1.28	2	1.28	5
Nausea	2.38	2	1.15	4	1.31	4
Worrying	2.01	3	1.09	5	1.80	3
Feeling Sad	1.81	4	1.08		1.92	2
Lack of Appetite	1.60	5	0.90		1.24	
Feeling Irritable	1.47		1.29	1	0.95	
Nervous	1.31		1.19	3	0.98	
Difficulty Breathing	-0.23		-1.86		1.93	1
Patients with breast cancer at time-point 2						
Lack of Energy	4.00	1	1.77	1	2.37	1
Nausea	1.69	2	1.03		0.06	
Feeling Bloated	1.46	3	0.81		0.66	
Chest Tightness	1.28	4	0.77		1.02	4
Pain	1.09	5	1.13	3	0.49	
Difficulty Sleeping	0.39		1.28	2	0.70	
Feeling Drowsy	0.34		1.13	4	0.76	
Difficulty Concentrating	0.39		1.03	5	0.57	
Difficulty Breathing	-0.14		0.35		2.01	2
Worrying	0.72		0.86		1.78	3
Feeling Sad	0.10		0.93		1.00	5
Patients with breast cancer at time-point 3						
Difficulty Concentrating	2.77	1	1.47	1	1.59	3
Nausea	2.66	2	0.04		0.28	
Abdominal Cramps	1.90	3	1.39	3	0.50	
Difficulty Breathing	1.64	4	0.66		2.33	1
Lack of Appetite	1.07	5	-0.29		0.94	
Feeling Bloated	1.05		1.44	2	0.15	
Difficulty Sleeping	0.22		1.08	4	-0.12	
Numbness or Tingling in Hands or Feet	0.81		1.07	5	0.44	
Feeling Sad	-0.25		0.60		1.70	2
Weight Gain	-0.03		0.83		1.41	4
Nervous	0.72		0.99		1.40	5

Supplemental Figures S1-S2-S3-S4-S5-S6

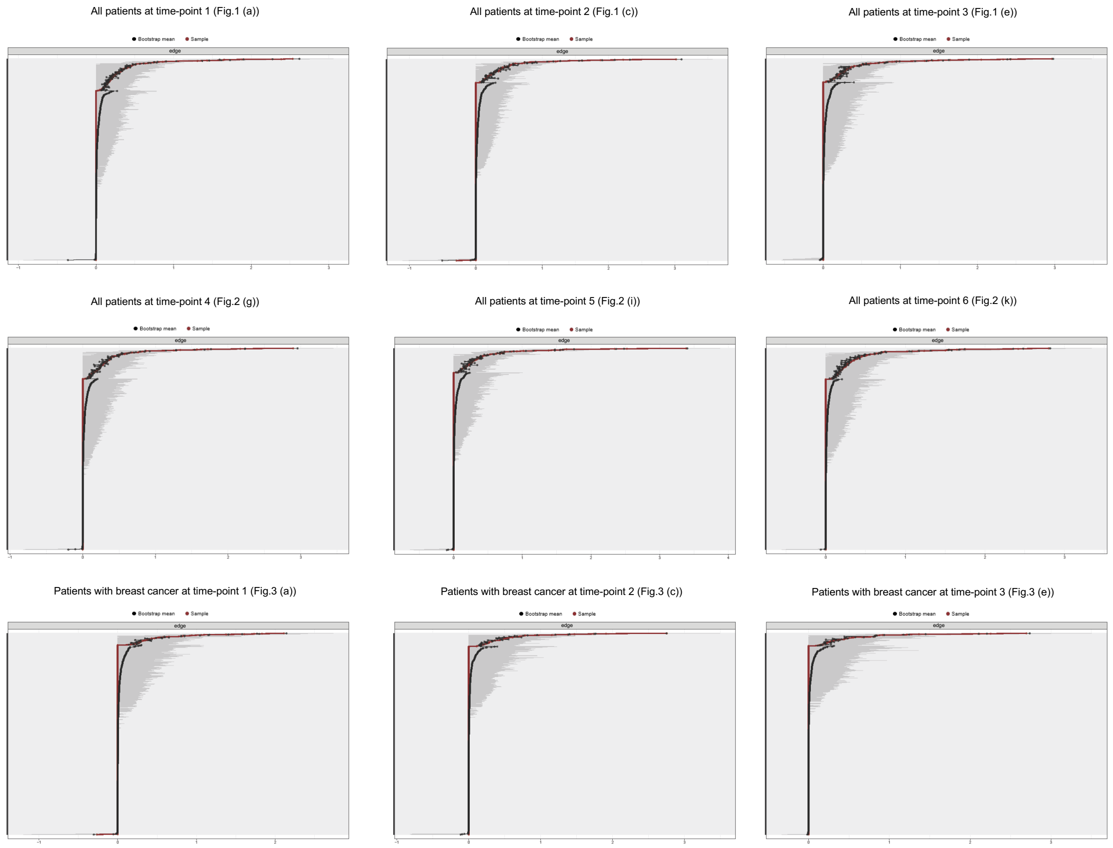

Figure S1 | Bootstrap 95% confidence intervals for estimated edge weights for the network of the 38 cancer symptoms over time and across cancer types. The edge weights, each horizontal line representing one edge, are represented by the red and black lines for sample values and mean of the bootstrap samples, respectively. The gray area indicates the 95% confidence intervals.

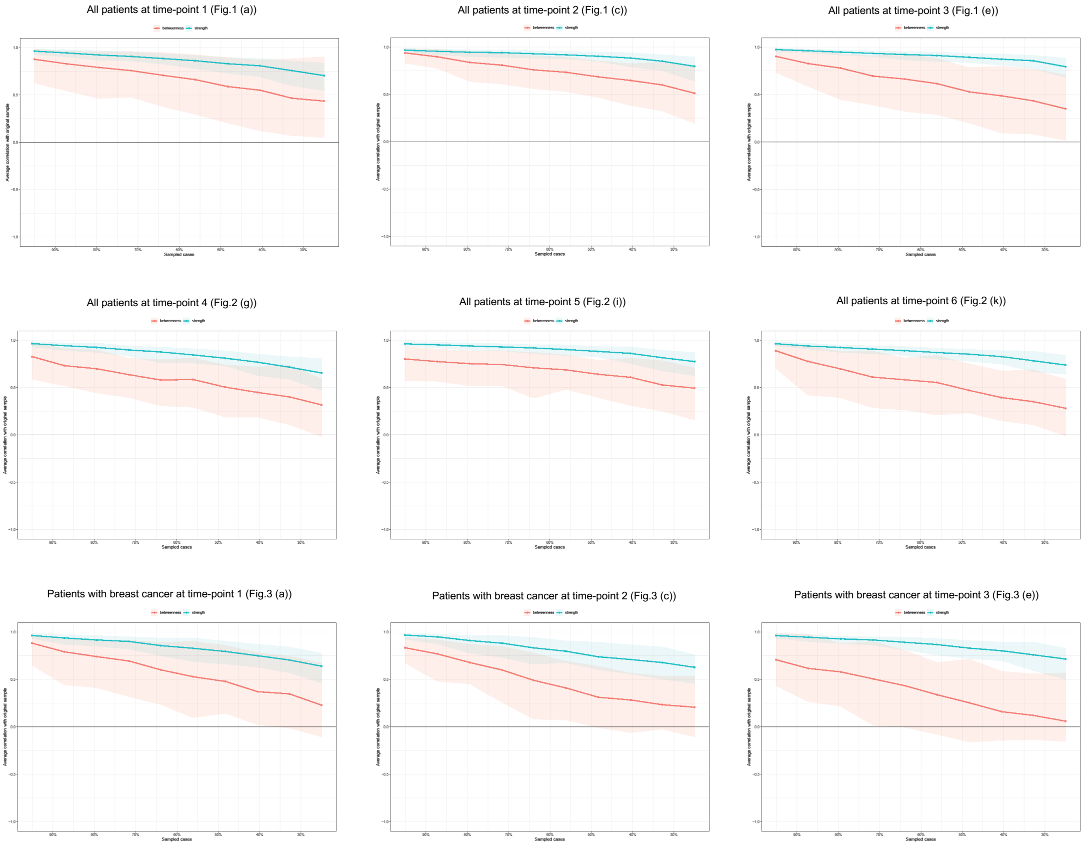

Figure S2 | Average correlations between the centrality indices of the networks of the 38 cancer symptoms over time and across cancer types sampled with cases dropped and the original sample. Lines indicate the means and areas indicate the range from the 2.5th quantile to the 97.5th quantile. The statistic closeness does not contain any variance and is therefore not shown.

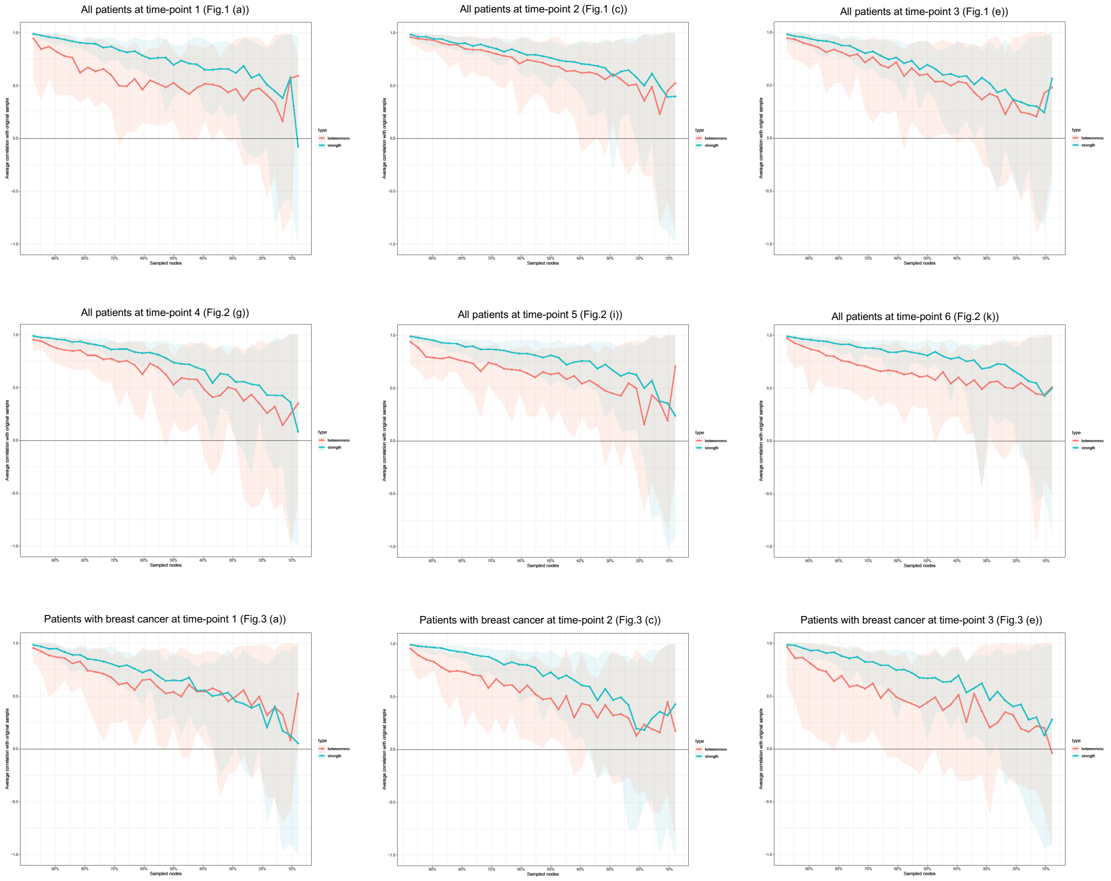

Figure S3 | Average correlations between the centrality indices of the networks of the 38 cancer symptoms over time and across cancer types sampled with nodes dropped and the original sample. Lines indicate the means and areas indicate the range from the 2.5th quantile to the 97.5th quantile. The statistic closeness does not contain any variance and is therefore not shown.

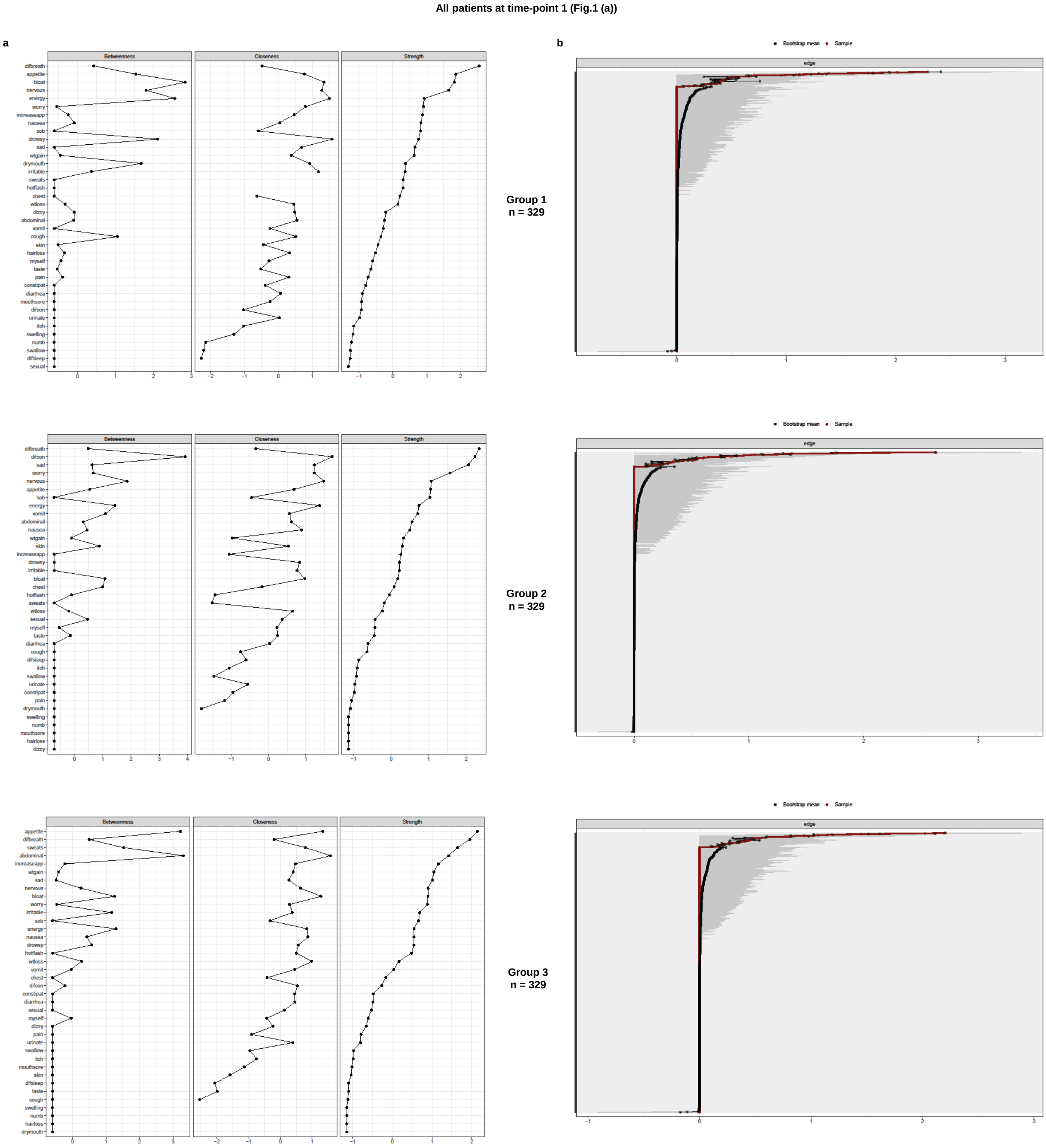

Figure S4 | a Centrality indices (betweenness, closeness, and strength) of the networks of the 38 cancer symptoms in each of 3 equally divided groups, across time point 1 shown in Fig.1 (a). **b** Bootstrap 95% confidence intervals for estimated edge weights for the network of the 38 cancer symptoms in each of 3 equally divided groups, across time point 1 shown in Fig.1 (a). The edge weights, each horizontal line representing one edge, are represented by the red and black lines for sample values and mean of the bootstrap samples, respectively. The gray area indicates the 95% confidence intervals.

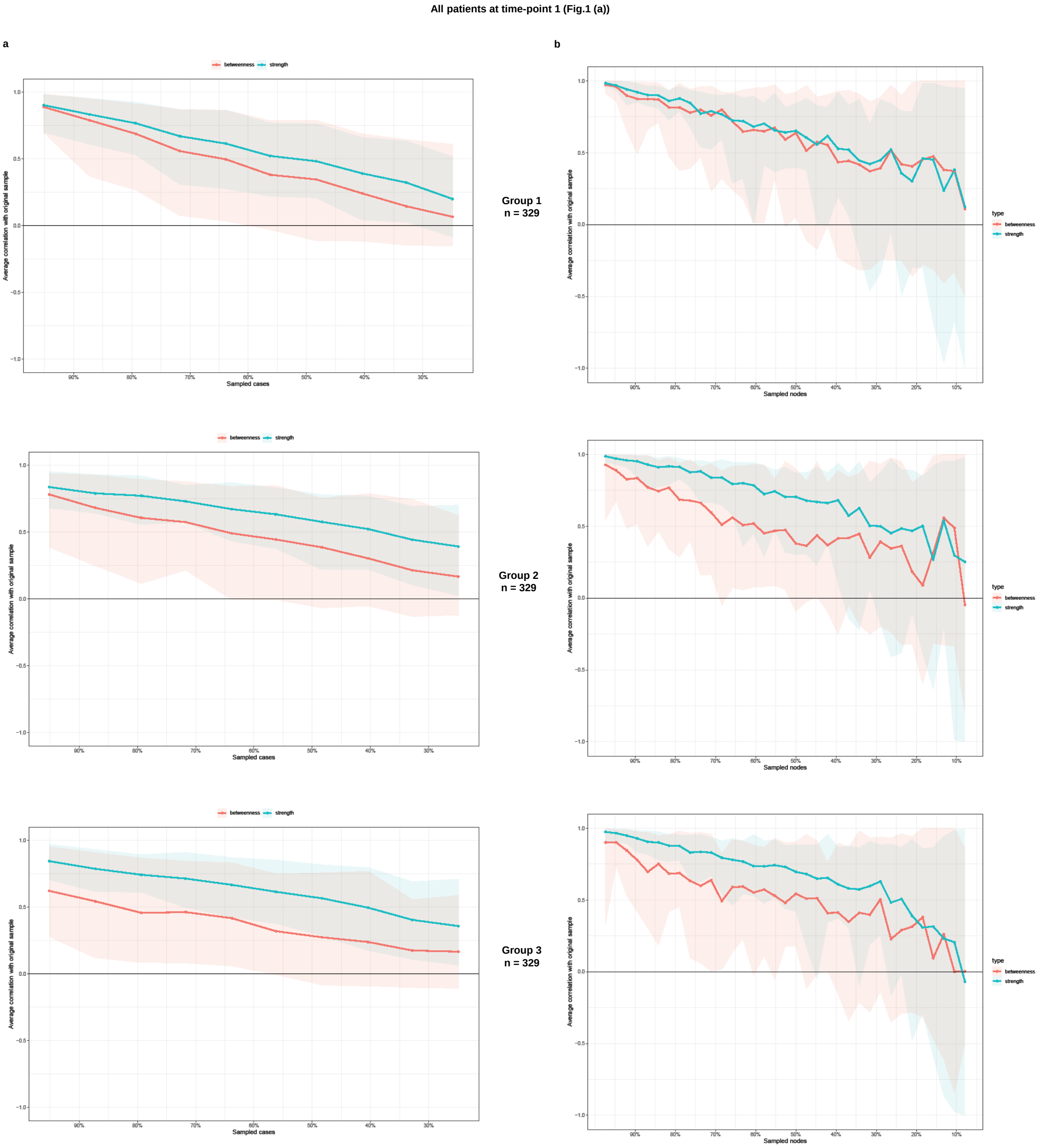

Figure S5 | a Average correlations between the centrality indices of the networks of the 38 cancer symptoms in each of 3 equally divided groups, across time point 1 shown in Fig.1 (a) sampled with cases dropped and the original sample. Lines indicate the means and areas indicate the range from the 2.5th quantile to the 97.5th quantile. The statistic closeness does not contain any variance and is therefore not shown. **b** Average correlations between the centrality indices of the networks of the 38 cancer symptoms in each of 3 equally divided groups, across time point 1 shown in Fig.1 (a) sampled with nodes dropped and the original sample. Lines indicate the means and areas indicate the range from the 2.5th quantile to the 97.5th quantile. The statistic closeness does not contain any variance and is therefore not shown.

Networks for patients with breast cancer (20 symptoms) Figure S6 (a) shows the network of 20 symptoms at time-point 1. All the connections are positive and it had a medium density (23.15%). Among all of the symptoms, hot flashes and sweats, feeling sad and worrying, lack of energy and feeling drowsy, worrying and feeling nervous, and feeling sad and feeling irritable were strongly correlated in pairs. The symptoms grouped into four clusters: psychological [shown in bisque], gastrointestinal [shown in green], hormonal [shown in blue], and sickness behaviour [shown in yellow]. In terms of centrality indices at time-point 1, for patients with breast cancer (see Figure S6 (b)), lack of energy ($r_b = 37, r_c = 0.01, r_s = 3.27$) had the highest values for betweenness and closeness scores, and feeling sad ($r_b = 11, r_c = 0.01, r_s = 3.56$), worrying ($r_b = 14, r_c = 0.01, r_s = 3.34$), and lack of energy had the highest strength scores. Therefore, our findings suggest that symptoms within the psychological and gastrointestinal clusters among the patients with breast cancer warrant clinical evaluation and management.

Figure S6 (c) illustrates the network of symptoms at time-point 2. All the connections were positive and it had a medium density (21.58%). Among all of the symptoms, hot flashes and sweats, feeling sad and worrying, lack of energy and feeling drowsy, lack of energy and difficulty concentrating, lack of energy and nausea were strongly correlated in pairs. The symptoms grouped into four clusters: psychological [shown in bisque], gastrointestinal [shown in green], hormonal [shown in blue], and sickness behaviour [shown in yellow]. In terms of the centrality indices at time-point 2 (see Figure S6 (d)), lack of energy had the highest scores across all three indices ($r_b = 80, r_c = 0.01, r_s = 4.46$). Therefore, sickness behaviour cluster may warrant comprehensive assessments and tailored interventions.

Figure S6 (e) illustrates the network of symptoms at time-point 3. All of the connections were positive and it had a medium density (21.05%). Among all of the symptoms, feeling sad and worrying, feeling sad and feeling irritable, nausea and lack of appetite, difficulty sleeping and difficulty concentrating, and lack of energy and feeling drowsy were strongly correlated in pairs. The symptoms grouped into four clusters: psychological [shown in bisque], gastrointestinal [shown in green], hormonal [shown in blue], and sickness behaviour [shown in yellow]. Concerning centrality indices at time-point 3 (see Figure S6 (f)), feeling irritable ($r_b = 39, r_c = 0.01, r_s = 3.15$) had the highest betweenness and closeness scores, and feeling sad ($r_b = 29, r_c = 0.01, r_s = 4.09$), difficulty concentrating ($r_b = 16, r_c = 0.01, r_s = 3.36$), and feeling irritable had the highest strength scores. Therefore, symptoms within the psychological, and sickness behaviour clusters warrant additional assessment and appropriate management.

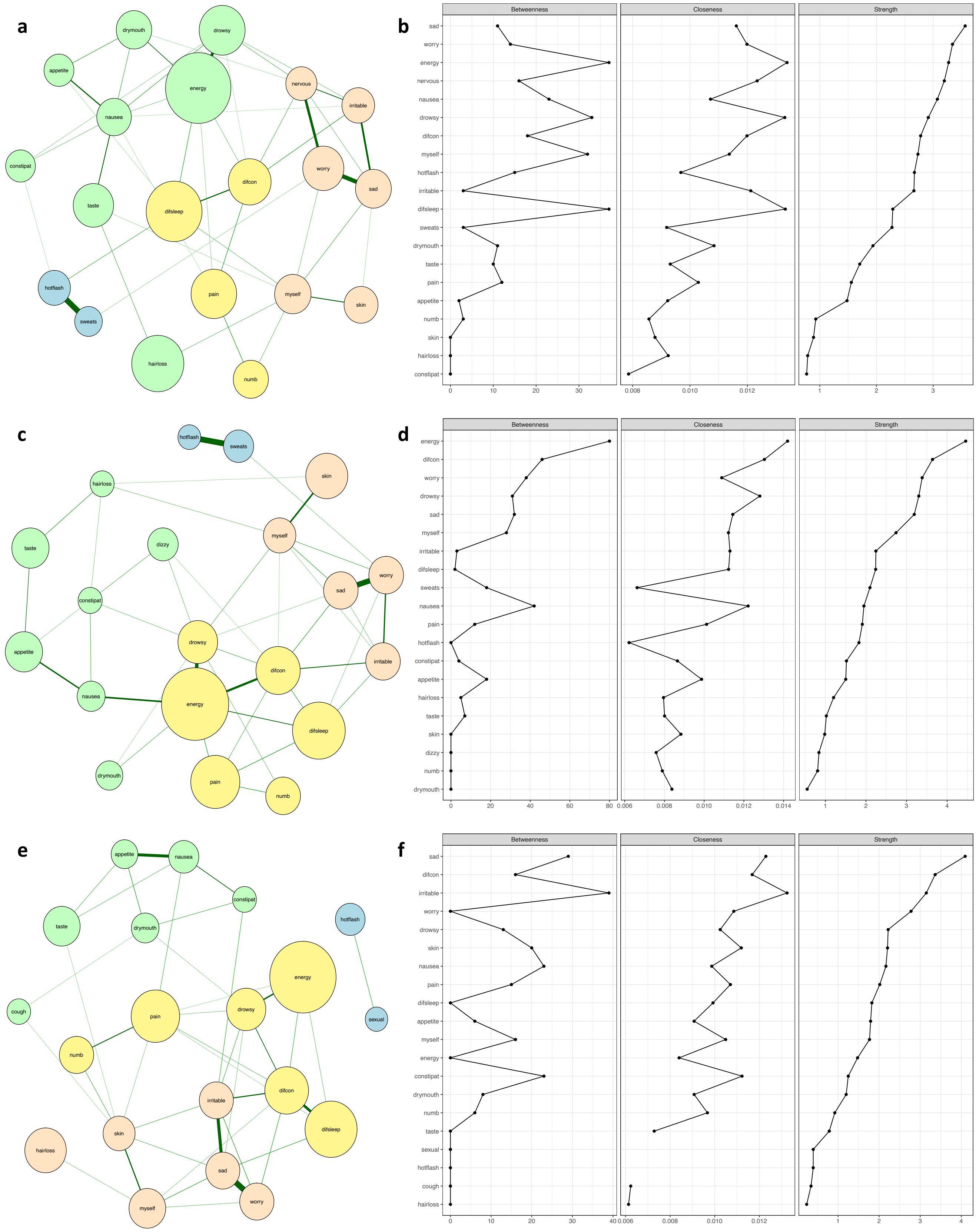

Figure S6 | The estimated networks for 20 cancer symptoms, most frequently occurring, with the identified symptom clusters and centrality indices during cycle 1 of CTX using ratings of symptom occurrence. Patients with "breast" cancer with complete data across the six assessments (n = 408) were included in these analyses. Nodes represent symptoms and edges represent pairwise correlations between the symptoms, after conditioning on all of the other nodes in the network. Symptom clusters are depicted with different colours. Centrality indices were ordered by strength values. Symptom(s) with no closeness coefficient appeared separated from the rest of the network. **a** Estimated network for 20 cancer symptoms with the identified clusters for time-point 1: prior to the second or third cycle of CTX administration. **b** Centrality indices (betweenness, closeness, and strength) for the estimated network shown in a. **c** Estimated network for 20 cancer symptoms with the identified clusters for time-point 2: approximately 1 week after CTX administration. **d** Centrality indices (betweenness, closeness, and strength) for the estimated network shown in c. **e** Estimated network for 20 cancer symptoms with the identified clusters for time-point 3: approximately 2 weeks after CTX administration. **f** Centrality indices (betweenness, closeness, and strength) for the estimated network shown in e.
